# CLRe: A Synergistic Dual‐Engine Framework for One‐Step Retrosynthesis Prediction

**DOI:** 10.1002/advs.76827

**Published:** 2026-07-29

**Authors:** Tianhao Su, Xitao Wang, Musen Li, Guanhua Qin, Shunbo Hu, Tong‐Yi Zhang

**Affiliations:** ^1^ Material Genome Institute Institute for the Conservation of Cultural Heritage Institute for Quantum Science and Technology Shanghai University Shanghai China; ^2^ Shanghai Xin Chang Rong Semiconductor Materials Co., Ltd. Shanghai China; ^3^ Key Laboratory of Silicate Cultural Heritage Conservation (Shanghai University) Ministry of Education Shanghai China; ^4^ Institute for the Conservation of Cultural Heritage Key Laboratory of Silicate Cultural Relics Conservation (Ministry of Education) Shanghai University Shanghai China

**Keywords:** contrastive learning, curriculum learning, retrosynthesis prediction, self‐supervised learning

## Abstract

One‐step retrosynthesis prediction is fundamentally limited by the random training order of sequence‐to‐sequence models and the inherent mismatch between local text generation and global chemical topology. Here we present **CLRe** (**C**ontrastive curriculum **L**earning for **Re**trosynthesis), a framework that integrates self‐supervised curriculum learning with topological buffering to resolve these bottlenecks. We introduce a label‐free contrastive metric that quantifies intrinsic molecular complexity to optimize training pacing. Furthermore, we adapt label smoothing to act as a topological buffer, which preserves the search entropy required for complex multi‐path chemical reasoning. We demonstrate that CLRe consistently improves performance on the USPTO‐50K and USPTO‐MIT datasets, significantly reducing accuracy disparities across historically challenging reaction classes. By capturing fine‐grained structural complexity orthogonal to standard reaction rules, CLRe offers a robust strategy for bridging data‐driven sequence generation with intrinsic chemical intuition.

## Graphical abstract

CLRe uses a contrastive difficulty score to order pretrained seq2seq fine‐tuning for retrosynthesis. Reaction embeddings define the ranking score, and a cumulative easy‐to‐hard schedule expands from the easiest subset to the full training set while earlier examples remain active. Matched USPTO‐50K evaluations show improved top‐k precursor‐set prediction, including on the globally hardest 5% test reactions.

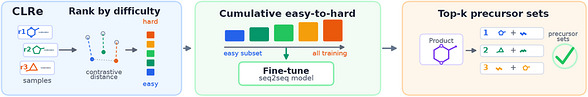

## 1 Introduction

Retrosynthesis analysis is one of the fundamental problems in organic chemistry, with the goal of deducing reactant combinations capable of synthesizing a given target product molecule [1]. Since Corey proposed the concept in 1969 [2], this problem has long relied on the empirical knowledge of organic chemists and manually designed transformation rules. Early computer‐aided retrosynthesis systems achieved automated reasoning by encoding expert rules [3, 4]. Still, such methods were strictly limited by the coverage of rule libraries and struggled to handle novel reaction types outside their encoded domains.

In recent years, the rapid advancement of deep learning has propelled data‐driven retrosynthesis from template‐based retrieval systems to end‐to‐end generative models. Traditional template‐based approaches rely on subgraph isomorphism and expert‐crafted reaction rules, which fundamentally suffer from poor scalability and an inability to generalize to out‐of‐distribution reaction spaces [5, 6, 7, 8]. To overcome these bottlenecks, the research paradigm has increasingly shifted toward template‐free sequence‐to‐sequence (seq2seq) architectures [9, 10, 11]. By representing chemical structures as linear strings (e.g., SMILES [12]) and treating retrosynthesis as a machine translation task, seq2seq models—particularly those empowered by the Transformer architecture [13]—have demonstrated exceptional capability in capturing long‐range atomic dependencies and complex structural transformations [14, 15]. Complementary to pure string models, graph‐based retrosynthesis frameworks have been developed to explicitly model disconnections and reaction centers [16, 17, 18, 19]. More recently, large‐language‐model (LLM) based retrosynthesis systems have emerged as a complementary direction, including instruction‐tuned dual‐task frameworks and large‐scale generative retrosynthesis models [20, 21, 22]. These approaches provide useful context for the current competitive landscape, but they differ substantially from the present study in model family, training setup, and evaluation protocol. Accordingly, our focus here is the controlled optimization of a pretrained seq2seq backbone rather than a cross‐paradigm comparison of all retrosynthesis architectures.

More recently, inspired by paradigm shifts in natural language processing, the “pretrain‐then‐finetune” strategy has demonstrated immense potential in cheminformatics. Pretrained Chemical Language Models (CLMs) [23, 24, 25, 26] leverage self‐supervised learning over massive, unlabeled chemical corpora to implicitly acquire fundamental physicochemical rules, such as atomic valency, stereochemical constraints, and general reactivity patterns [27, 28, 29]. As a state‐of‐the‐art representative of this trend, ReactionT5 [30] was pretrained specifically on large‐scale reaction datasets. By establishing a robust understanding of structural transformations prior to task‐specific fine‐tuning, such foundation models significantly mitigate data sparsity issues, yielding substantial improvements in both predictive accuracy and generation validity.

Despite these remarkable advances, existing fine‐tuning paradigms suffer from two fundamental bottlenecks that severely restrict their performance on highly complex chemical transformations. First, at the macroscopic optimization level, they adopt a randomly shuffled training order, treating all chemical reactions equally. In reality, the prediction difficulty of reactions varies significantly. Randomly exposing models to highly complex cascade rearrangements in the early stages of training generates severe gradient noise and destabilizes convergence. While Curriculum Learning (CL) [31, 32, 33, 34] offers a theoretical solution by organizing materials from “easy to hard,” its application in chemistry is hindered by the lack of a label‐free, computationally efficient metric to quantify the context‐dependent difficulty of molecular transformations. Static structural proxies (e.g., synthetic accessibility scores [35]) often diverge from the actual optimization difficulty encountered by neural networks. Second, at the microscopic architectural level, a profound representational gap exists between the autoregressive nature of CLMs and the global topological requirements of chemical reasoning. Text‐based sequence models rely on local, next‐token prediction mechanisms. However, complex chemical decisions—such as determining which protecting group reagent to introduce, or identifying the correct regioselective site for oxidation—are inherently global, intent‐driven tasks. Hard‐label optimization violently penalizes the model during these macroscopic reagent retrievals or topological string reorderings, forcing the model into autoregressive myopia and causing catastrophic pruning of chemically viable paths during beam search decoding [36, 37].

To systematically address these interrelated bottlenecks, we propose **CLRe** (**C**ontrastive curriculum **L**earning for **Re**trosynthesis), a synergistic dual‐engine optimization framework. Engine A (Macroscopic Pacing): We introduce a purely data‐driven, self‐supervised difficulty metric based on contrastive representation learning to govern the pacing of training data. We hypothesize that if a molecule's topological features are difficult for an encoder to map into an invariant latent space across augmented string views, it possesses highly unusual structural motifs, making its retrosynthetic pathway correspondingly harder to deduce. This “unfamiliarity” score naturally circumvents the biases of static heuristics. Building on this signal, we design a modular curriculum pipeline that couples a linear pacing function with a curriculum‐aware adaptive early stopping mechanism to prevent premature termination during exploration phases. Engine B (Microscopic Buffering): To resolve the text‐graph epistemological conflict, we redefine Label Smoothing (LS) (ε=0.05) not merely as a generic regularizer, but as a *Topological and Intent Buffer*. By integrating LS within our curriculum framework, we mathematically soften the extreme penalties inflicted by hard labels during topology‐induced SMILES string reorderings. This grants the beam search algorithm the critical entropy needed to sustain multi‐path exploration, preventing the model from collapsing when forced to make global chemical decisions.

## 2 Methods

In this section, we detail the proposed CLRe framework. We first formulate the one‐step retrosynthesis problem as a sequence generation task, then introduce our self‐supervised contrastive approach for quantifying reaction difficulty, and finally present the complete curriculum learning pipeline, which integrates a linear pacing function, an adaptive early stopping mechanism, and label smoothing regularization.

### 2.1 Problem Formulation

One‐step retrosynthesis prediction can be formalized as a conditional sequence‐to‐sequence generation problem. Given a target product molecule represented by a SMILES string p, the objective is to predict the corresponding reactant combination r capable of synthesizing the product. In standard chemical datasets (e.g., USPTO), reactants are typically concatenated using dot‐separated SMILES strings (e.g., reactant1.reactant2.reactant3). The conditional probability of generating the reactant sequence is factored autoregressively as:

(1) $$P\left(r|p\right)=\prod_{t=1}^{T}P\left(r_{t}|r_{<t},p\right)$$

where $r_{t}$ is the t‐th token in the reactant SMILES, $r_{<t}$ denotes the prefix sequence generated before position t, and T is the total length of the target reactant sequence. Following the architecture of pretrained chemical language models like ReactionT5 [30], we prepend a task‐specific prompt to the product p. During fine‐tuning, the network parameters θ are optimized to maximize the log‐likelihood of the ground‐truth reactant sequences over the training corpus:

(2) $$L_{\text{MLE}}=-\sum_{i=1}^{N}\log P\left(r^{\left(i\right)}|p^{\left(i\right)};\theta \right)$$

where N represents the total number of training samples.

### 2.2 Self‐Supervised Contrastive Difficulty Assessment

A central bottleneck in implementing curriculum learning for molecular tasks is the accurate quantification of sample difficulty. Traditional methods relying on heuristic indicators [35] often fail to capture the dynamic, task‐specific learning complexity encountered by neural networks. To address this, CLRe introduces a purely data‐driven, self‐supervised contrastive learning approach that quantifies molecular difficulty by evaluating the “irregularity” of a molecule within the latent representation space.

#### 2.2.1 The Familiarity Hypothesis

Our difficulty metric is grounded in the *Familiarity Hypothesis*: If a molecule's SMILES representation is difficult for a contrastive encoder to correctly align with its augmented views, the molecule possesses more unusual topological features. Consequently, deducing its retrosynthetic pathway is inherently more challenging for generative models.

Fundamentally, this metric elegantly bridges the underlying data distribution of chemical space with intrinsic chemical intuition. In real‐world reaction databases like USPTO, the data distribution is heavily skewed, exhibiting a pronounced long‐tail phenomenon. The high‐density regions of this distribution are dominated by highly standardized, modular transformations (e.g., amide couplings or Suzuki cross‐couplings) acting on ubiquitous scaffolds. Because the contrastive encoder observes abundant variations of these common motifs, it easily learns smooth, invariant representations, yielding a remarkably low InfoNCE loss. From a chemical perspective, these low‐loss molecules correspond to robust synthetic methodologies with obvious disconnection sites (synthons) and abundant commercially available precursors, making their one‐step retrosynthesis naturally “easy.”

Conversely, the long‐tail (sparse) regions of the distribution comprise structurally anomalous molecules with dense stereocenters, highly strained bridged or fused ring systems, and complex natural product‐like topologies. Lacking sufficient structural neighbors in the training manifold, the encoder struggles to align their augmented views, resulting in a significantly higher contrastive loss. This data‐driven “unfamiliarity” perfectly mirrors the cognitive difficulty experienced by expert chemists: molecules residing in these sparse chemical spaces typically lack trivial commercial precursors and often demand highly specific, non‐intuitive cascade reactions or intricate skeletal rearrangements. By capturing this underlying distribution density, the contrastive loss naturally and quantitatively reflects the true synthetic complexity.

#### 2.2.2 Representation Learning via SMILES Randomization

Contrastive learning isolates discriminative representations by pulling positive sample pairs closer and pushing negative sample pairs apart in a latent manifold [38]. To construct valid positive pairs for a given molecule without altering its underlying chemical graph, we leverage SMILES randomization augmentation following Bjerrum's SMILES enumeration strategy and the randomized‐SMILES retrosynthesis protocol of Tetko, Karpov, Van Deursen, and Godin, implemented with RDKit [11, 39, 40].

For each target product molecule $p^{\left(i\right)}$, we generate two chemically equivalent but syntactically distinct SMILES representations, $p_{i}^{1}$ and $p_{i}^{2}$, by randomly permuting the traversal order of the heavy atoms. This non‐destructive augmentation forces the model to learn permutation‐invariant, structure‐intrinsic molecular features. The paired string views are subsequently fed into a lightweight Transformer encoder. The encoder comprises a token embedding layer ($d_{\text{model}}=256$), 4 Transformer layers featuring multi‐head self‐attention (8 heads, feed‐forward dimension 1024), and a mean pooling layer followed by a 2‐layer multi‐layer perceptron (MLP) projection head. The final output is a 128‐dimensional normalized vector. With only ∼2M parameters, this auxiliary encoder is computationally negligible to train compared to the primary sequence‐to‐sequence model.

#### 2.2.3 Symmetric InfoNCE Loss and Difficulty Scoring

The contrastive encoder maps the augmented string views $\left(p_{i}^{1},p_{i}^{2}\right)$ into dense latent representations $\left(z_{i}^{1},z_{i}^{2}\right)$. The encoder is optimized using the symmetric InfoNCE loss [41]. For a given mini‐batch of B samples, the objective function is defined as:

(3) $$L_{\text{InfoNCE}}=\frac{1}{2B}\sum_{i=1}^{B}\left[\ell \left(z_{i}^{1},z_{i}^{2}\right)+\ell \left(z_{i}^{2},z_{i}^{1}\right)\right]$$

where $\ell (z_{i}^{a},z_{i}^{b})$ denotes the directional InfoNCE loss:

(4) $$\ell \left(z_{i}^{a},z_{i}^{b}\right)=-\log\frac{\exp(\text{sim}\left(z_{i}^{a},z_{i}^{b}\right)/\tau )}{\sum_{k=1}^{B}\sum_{v\in \{1,2\}}1_{\left[(k,v)\neq (i,a)\right]}\exp\left(\text{sim}\left(z_{i}^{a},z_{k}^{v}\right)/\tau \right)}$$

Here, $\text{sim}\left(u,v\right)=u^{⊤}v/\left(∥u∥∥v∥\right)$ represents the cosine similarity, τ=0.07 is the temperature hyperparameter controlling the sharpness of the distribution, and $1_{\left[(k,v)\neq (i,a)\right]}$ is an indicator function excluding the anchor sample itself.

Upon convergence of the contrastive encoder, we utilize the specific instance‐level InfoNCE loss as the **contrastive difficulty score**. For each product $p^{\left(i\right)}$ in the training set, we calculate $\ell (z_{i}^{1},z_{i}^{2})$ via a single deterministic forward pass. The raw divergence scores are then min‐max normalized to establish a continuous difficulty spectrum $d^{\left(i\right)}\in \left[0,1\right]$:

(5) $$d^{\left(i\right)}=\frac{\ell^{\left(i\right)}-\min_{j}\ell^{\left(j\right)}}{\max_{j}\ell^{\left(j\right)}-\min_{j}\ell^{\left(j\right)}}$$

**Static Pre‐calculation and Strict Data Isolation**. To prevent dynamic computational overhead during the main sequence‐to‐sequence fine‐tuning phase, all difficulty scores $d^{\left(i\right)}$ are statically pre‐calculated and cached. Furthermore, to rigorously prevent data leakage, the contrastive encoder is trained *exclusively* on the training split of the dataset. Validation and test sets remain completely sequestered during both the representation learning and the difficulty scoring phases, guaranteeing that the curriculum design is informed solely by knowledge available within the training boundaries.

### 2.3 The CLRe Curriculum Framework

With robust difficulty scores $d^{\left(i\right)}$ mathematically established, CLRe introduces a modular curriculum learning pipeline designed to progressively expose the generative model to training samples from structurally simple to highly irregular.

#### 2.3.1 Training Data Sorting and Linear Pacing

We sort the entire training corpus in ascending order based on their contrastive difficulty scores: $d^{\left(1\right)}\leq d^{\left(2\right)}\leq \cdots \leq d^{\left(N\right)}$. To regulate the velocity at which harder samples are introduced, we employ a linear pacing function. At training epoch t (0‐indexed), the proportion of accessible training data λ(t) is computed as:

(6) $$\lambda \left(t\right)=\min\left(1,\frac{t+1}{f\cdot T}\right)$$

where T represents the total number of maximum training epochs, and f∈(0,1] is the curriculum fraction hyperparameter controlling the pacing velocity. For instance, setting f=0.5 implies the model gains access to the complete dataset from the 50% milestone onwards. At epoch t, the model is strictly constrained to sample from the easiest ⌊λ(t)·N⌋ reactions. Intuitively, this procedure resembles a school curriculum. Early epochs expose the model mainly to elementary, standardized reactions; intermediate epochs unlock moderately complex cases; and the final stage introduces the hardest, topologically irregular reactions. Thus, the model progresses from simple to complex chemistry in a controlled graduation‐like cycle rather than being forced to solve all difficulty levels from the first epoch. Importantly, CLRe uses a cumulative easy‐to‐hard curriculum rather than replacing easy samples with hard samples. Training reactions are sorted by the CLRe difficulty score, and the active training subset expands linearly from the easiest prefix to the full training set. With curriculum fraction = 0.5, all training samples are introduced by the midpoint of a 300‐epoch schedule. Therefore, newly introduced hard reactions are added together with the previously learned easier reactions, which remain in the active subset and continue to regularize the model. Combined with the smaller late‐stage optimization steps from the learning‐rate schedule, this makes hard‐case adaptation closer to conservative fine‐tuning on progressively harder examples than to disruptive relearning from a new data distribution. This mechanism is intended to reduce the risk that hard examples overwrite easy‐case behavior, while still allowing the model to refine decision boundaries for difficult reactions.

#### 2.3.2 Curriculum‐Aware Adaptive Early Stopping

Standard early stopping monitors validation loss and terminates training if it stagnates for a predefined patience period. However, in a dynamic curriculum setting, the validation loss characteristically exhibits transient spikes when novel, highly irregular samples are newly introduced. Standard early stopping algorithms might mistakenly interpret these necessary exploration phases as catastrophic overfitting, triggering premature termination.

To circumvent this, CLRe employs a curriculum‐aware mechanism: during the active curriculum expansion stage (λ(t)<1), the early stopping counter is completely suspended. The patience counter only resumes monitoring the validation loss trajectory after the curriculum is fully deployed (λ(t)=1).

#### 2.3.3 Label Smoothing as a Topological and Intent Buffer

A critical yet heavily overlooked challenge in applying NLP architectures to chemical tasks is the fundamental misalignment between the local, autoregressive nature of next‐token prediction and the global, topological nature of chemical reasoning. Once the curriculum expands to include highly irregular, complex samples, the standard optimization objective inherently forces the model into premature convergence. To mitigate this and bridge the text‐graph epistemological gap, we integrate Label Smoothing (LS) [42, 43] as a structural buffer in the CLRe objective.

Standard cross‐entropy loss employs hard, one‐hot target vectors, commanding absolute certainty at every decoding step:

(7) $$L_{\text{CE}}=-\sum_{t=1}^{T}\log P\left(r_{t}=y_{t}|r_{<t},p\right)$$

While effective for simple functional group interpolations (e.g., esterification), this hard‐label objective is catastrophic for complex retrosynthetic tasks due to what we term *autoregressive myopia*. For instance, in protection reactions (Class 5), the model observes only a local protected motif in the product but must generate a long, independent reagent SMILES string (e.g., ${\mathrm{Boc}}_{2}O$ or TFA anhydride) based on global reaction intent. At the branching point of predicting the first character of this reagent, hard labels inflict extreme gradient penalties on any uncertainty. This forces the model to memorize the most frequent token rather than learning global intent mapping, leading to catastrophic pruning of correct paths during beam search.

Similarly, in regioselective reactions such as oxidations (Class 8), modifying a single double bond can induce substantial topology‐driven SMILES string reordering under RDKit canonicalization. Hard labels strictly penalize these isomorphic but string‐divergent exploration paths.

To resolve this, LS acts as a form of entropy maximization by replacing hard labels with soft target distributions:

(8) $$q\left(r_{t}|y_{t}\right)=\left(1-\varepsilon \right)\cdot \delta \left(r_{t}=y_{t}\right)+\frac{\varepsilon }{V}$$

where ε is the smoothing coefficient, V is the vocabulary size of the SMILES tokenizer, and δ represents the Dirac delta function. The smoothed loss function becomes:

(9) $$L_{\text{LS}}=\left(1-\varepsilon \right)L_{\text{CE}}+\varepsilon L_{\text{uniform}}$$

Given the ultra‐compact vocabulary size of SMILES (typically V≈200), the uniform distribution $\frac{\varepsilon }{V}$ allocates a highly concentrated, mathematically significant tolerance to non‐target tokens. In the specific context of CLRe, this ε acts as a crucial survival mechanism: it prevents the sequence model from collapsing under gradient polarization during macroscopic reagent retrieval, and cushions the penalization of topology‐induced SMILES string reorderings. By maintaining decoding entropy, LS enables beam search to keep multiple, chemically viable sub‐graphs alive simultaneously, thereby transforming a rigid text‐continuation model into a robust global chemical decision‐maker.

### 2.4 Training Details

We employ ReactionT5v2 as the base sequence‐to‐sequence generator, a T5‐base architecture comprising 198M parameters pretrained on extensive chemical reaction corpora. Fine‐tuning is conducted on the USPTO‐50K and USPTO‐MIT datasets.

All models are optimized using the AdamW optimizer with a learning rate of $5\times 10^{-5}$ and a weight decay of 0.01, combined with a linear learning‐rate warmup spanning the first 10% of training steps. Unless explicitly stated otherwise, we utilize a batch size of 64 per GPU (yielding an effective batch size of 128 across 2 GPUs), mixed precision training (FP16 via PyTorch AMP), 1 step of gradient accumulation, and a maximum sequence length of 128 tokens. Inference is executed using beam search with num_beams=10.

For the CLRe curriculum strategy, hyperparameters are set to a curriculum fraction f=0.5 (accessing the full dataset at 50% of total epochs), a label smoothing coefficient ε=0.05, and an early‐stopping patience of 20 epochs. For the contrastive encoder pretraining, we utilize a batch size of 256, a learning rate of $1\times 10^{-3}$, a temperature τ=0.07, and train for 100 epochs using the standard Adam optimizer. All experiments were conducted on a workstation equipped with 2× NVIDIA RTX PRO 6000 GPUs (96GB VRAM each).

## 3 Results and Discussion

### 3.1 Experimental Setup

#### 3.1.1 Datasets and Evaluation

We evaluate the proposed CLRe framework on two standard retrosynthesis benchmarks: USPTO‐50K [9] and the large‐scale USPTO‐MIT [44]. USPTO‐50K contains 50,016 reactions categorized into 10 distinct reaction classes, split into 40,513/4,501/5,002 for training, validation, and testing. To rigorously ensure that our evaluation measures genuine generalization rather than structural memorization, we conducted a data overlap analysis. The maximum Morgan fingerprint Tanimoto similarity (radius = 2) between the test and training sets is strictly bounded, confirming the absence of exact duplicate reactions. USPTO‐MIT provides a more formidable challenge with approximately 480K reactions, evaluating the framework's scalability. Model performance is systematically assessed using top‐k exact match accuracy (k=1,3,5,10) and SMILES validity. Because USPTO‐50K provides a single reference reactant set for many products, strict exact‐match top‐k accuracy can under‐credit chemically plausible alternative disconnections. To address this ambiguity, we report round‐trip relaxed accuracy as a complementary diagnostic: a prediction is counted as relaxed‐correct if it either matches the reference reactants or can be independently validated to regenerate the target product. This metric is used only as a supplement to canonical exact top‐k accuracy, which remains the primary literature‐comparable metric. Unless otherwise specified, inference is conducted using beam search with a width of 10. Detailed evaluations regarding the pretraining initialization are provided in the Supporting Information.

#### 3.1.2 Baselines and Fair Comparison Protocol

To ensure a rigorous evaluation, all baseline models were re‐implemented and trained under identical hardware conditions, random seeds, and strict hyperparameter budgets. We compare the complete CLRe framework (Dual‐Engine) against: (1) **Zero‐shot Inference** using the pretrained ReactionT5v2; (2) **Random Baseline**, representing the standard fine‐tuning paradigm with randomly shuffled data; (3) **Formula‐based CL** [45], utilizing a heuristic static complexity score (d=0.5×SA+0.3×Rings+0.2×HeavyAtoms); and (4) **Engine A Only**, utilizing contrastive pacing and curriculum early stopping, but omitting the topological buffer (Label Smoothing).

### 3.2 Main Results: Synergistic Performance on Benchmarks

#### 3.2.1 Performance on USPTO‐50K

Table 1 presents the comparative results on the USPTO‐50K test set. The standard Random Baseline achieves a top‐1 accuracy of 77.47%, highlighting the inherent limitations of treating all chemical transformations with equal optimization priority. Integrating Engine A (Contrastive Pacing) elevates the top‐1 accuracy to 89.86%, outperforming the heuristic Formula‐based CL (87.23%). This validates our hypothesis that data‐driven representational “unfamiliarity” provides a superior, context‐aware pacing signal compared to static molecular descriptors.

**TABLE 1:** Retrosynthesis prediction results on the USPTO‐50K test set. CLRe (Dual‐Engine) incorporates both Contrastive Pacing (Engine A) and the Topological Buffer (Engine B).

Method	Top‐1	Top‐3	Top‐5	Top‐10	Validity
Zero‐shot	34.57%	—	—	—	99.18%
Random Baseline	77.47%	87.39%	88.88%	90.00%	99.72%
Formula‐based CL	87.23%	92.36%	92.74%	93.06%	99.72%
Engine A Only (Contrastive Pacing)	89.86%	92.74%	93.22%	93.52%	99.76%
**CLRe (Dual‐Engine)**	**94.08%**	**96.20%**	**96.66%**	**96.94%**	**99.78%**

However, the true potential of the framework is unlocked when Engine A is synergistically coupled with Engine B (the Topological Buffer). Within this controlled ReactionT5v2‐based seq2seq fine‐tuning setting, the complete CLRe framework achieves the strongest performance observed in this study, with a **94.08%** top‐1 accuracy, yielding a formidable **+16.61pp** absolute improvement over the Random Baseline. Furthermore, the generated SMILES validity reaches 99.78%, confirming that the aggressive exploration encouraged by Engine B does not compromise the model's fundamental adherence to chemical syntax.

#### 3.2.2 Scope of Performance Comparison

The USPTO‐50K comparison in Table 1 is intentionally restricted to methods fine‐tuned under the same ReactionT5v2 backbone or closely matched heuristic curriculum settings. Therefore, the 94.08% top‐1 result should be interpreted as a controlled seq2seq fine‐tuning comparison rather than a universal leaderboard statement across all retrosynthesis paradigms. In particular, recently reported LLM‐based systems such as ChemDual, RSGPT, and RetroDFM‐R [20, 21, 22] differ substantially in model scale, supervision strategy, and evaluation framing, and are cited here primarily to contextualize the broader competitive landscape.

#### 3.2.3 Resolving Autoregressive Myopia: Per‐Class Analysis

To deeply understand the origin of this performance surge, we dissect the top‐1 accuracy across the 10 distinct reaction classes (see Table S2). Under standard random training, sequence‐to‐sequence models exhibit severe performance polarization: they excel at localized, predictable transformations (e.g., Class 1: 82.26%) but catastrophically fail on tasks requiring complex reagent generation or massive SMILES string reordering, such as Class 5 (Protection, 40.00%) and Class 9 (Functional group interconversion, 72.84%).

The implementation of the CLRe Dual‐Engine framework fundamentally alters this optimization landscape. The most profound gains manifest precisely in these historically challenging categories: Class 5 experiences an unprecedented surge of **+54.29pp** (reaching 94.29%), and Class 3 (C–N bond formation) improves by **+26.42pp**.

**FIGURE 1:**
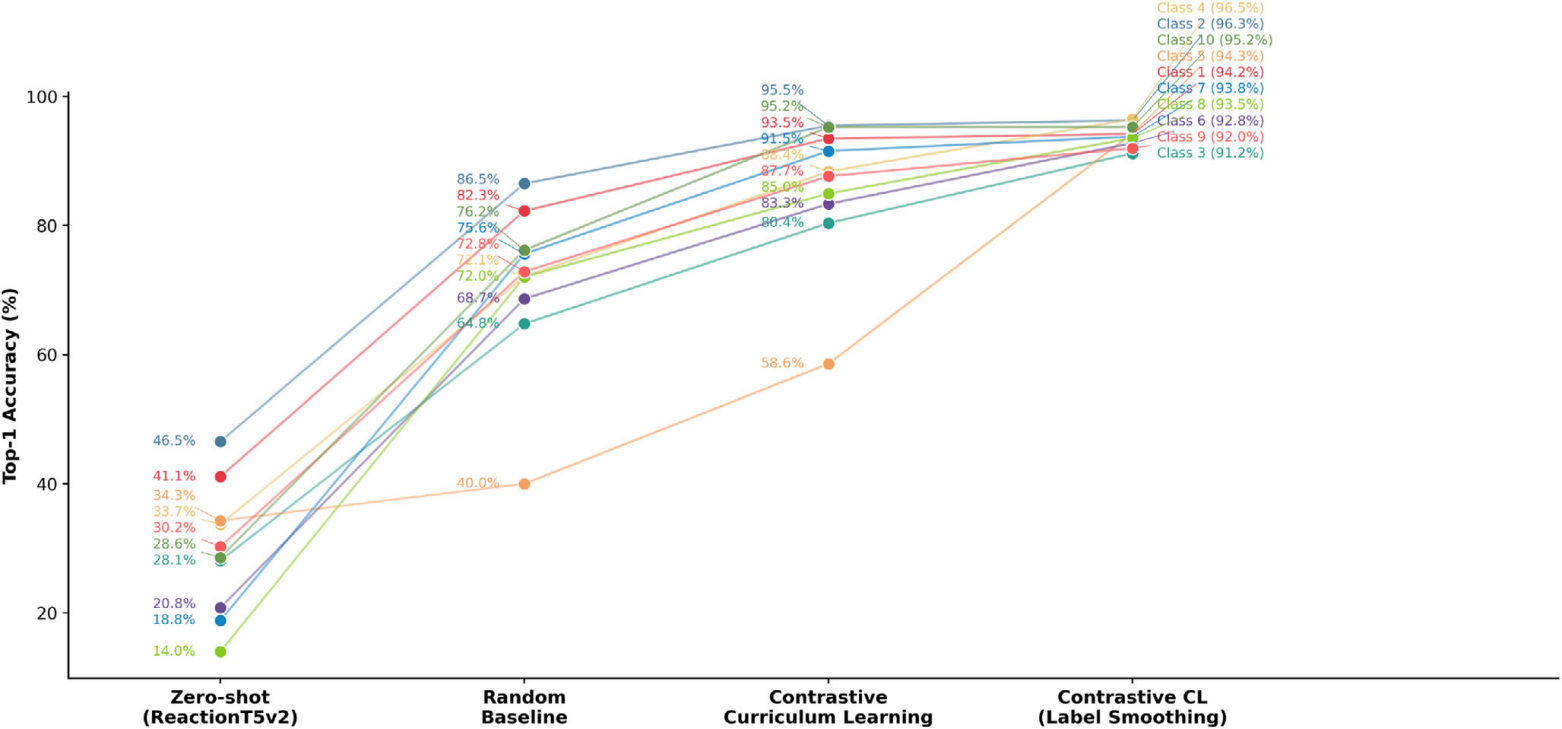
Top‐1 Accuracy Trajectory across the 10 reaction classes. The application of Contrastive Curriculum Learning, and particularly Label Smoothing, dramatically lifts the underperforming classes (e.g., Class 5, bottom curve of the Random Baseline), compressing the massive variance seen in standard training into a tight, high‐performance cluster.

Figure 1 visually encapsulates this paradigm shift. Under standard training (Random Baseline), the model exhibits severe performance divergence across classes. However, as the Dual‐Engine framework is sequentially applied, the trajectory of historically challenging classes (e.g., Class 5 and Class 3) sharply converges upward. The framework systematically mitigates the “autoregressive myopia” of text‐based models, narrowing the severe inter‐class performance disparity from **46.51pp** down to a tightly clustered **5.32pp**.

To test whether CLRe improves genuinely difficult reactions, we performed a post‐hoc hardest‐case analysis on USPTO‐50K. We ranked all test reactions by the CLRe contrastive difficulty score and selected the globally hardest 5% subset, yielding 251 hard test cases. This subset covers all 10 reaction classes, indicating that it is not dominated by a single class. Under the stricter beam = 10 exact‐string protocol, CLRe LS = 0.05 improved Top‐1 accuracy on this hard subset from 77.69% for the random baseline to 93.63%, corresponding to a +15.94 percentage‐point gain. The conclusion remained consistent under a more relaxed beam = 20 canonical‐match sensitivity check, where CLRe improved Top‐1 accuracy from 80.48% to 94.42%. Full per‐class hard‐case distributions and top‐k results under both matching protocols are provided in Section S4.

#### 3.2.4 Scalability: Performance on USPTO‐MIT

To ascertain the robustness and scalability of the CLRe framework in data‐rich environments, we evaluated it on the massive USPTO‐MIT dataset (∼480K reactions). As shown in Table 2, deploying Engine A (Contrastive Pacing) alone elevates the top‐1 accuracy from 77.09% to 79.46% (+2.37pp). While this confirms the scalability of macroscopic pacing, the relative gain is conservative. This aligns with optimization theory: the sheer abundance of diverse supervision signals in massive corpora inherently dampens early gradient variance, thereby compressing the marginal utility of pacing alone.

**TABLE 2:** Retrosynthesis prediction results on the large‐scale USPTO‐MIT test set. The full Dual‐Engine implementation shatters the performance ceiling, proving its necessity even in data‐rich regimes.

Method	Top‐1	Top‐3	Top‐5	Top‐10	Validity
Random Baseline	77.09%	86.53%	88.30%	89.60%	**99.94%**
Engine A Only	79.46%	87.27%	88.73%	90.02%	**99.94%**
**CLRe (Dual‐Engine)**	**91.76%**	**95.35%**	**95.81%**	**96.08%**	99.87%

However, the fundamental text‐graph epistemological conflict remains unresolved by pacing. When we fully deploy the synergistic Dual‐Engine framework (integrating the LS = 0.05 Topological Buffer), the performance experiences a massive surge, reaching an extraordinary **91.76%** top‐1 accuracy. This represents a formidable **+14.67pp** absolute improvement over the standard Random Baseline. This breakthrough on a half‐million reaction corpus definitively proves that mitigating “autoregressive myopia” via topological buffering is not merely a small‐data regularization trick, but a fundamental architectural imperative for chemical sequence models.

### 3.3 Engine A Analysis: Decoding Macroscopic Pacing

The performance surge generated by Engine A relies entirely on the quality of the self‐supervised contrastive difficulty metric. To rigorously validate why this metric outperforms traditional heuristics, we dissect its optimization behavior, pacing dynamics, and statistical properties.

#### 3.3.1 Isolating the Difficulty Metric

Table 3 isolates the difficulty assessment module while keeping the linear pacing and early stopping mechanisms fixed (Engine B omitted). We evaluate three paradigms: (1) **Formula‐based**, representing static structural heuristics; (2) **Model Loss‐based**, a conventional ML approach requiring an expensive pretrained teacher model to score samples; and (3) **Contrastive (ours)**, representing the label‐free representational “unfamiliarity.”

**TABLE 3:** Ablation of difficulty assessment mechanisms on USPTO‐50K (Linear pacing, no LS).

Difficulty Metric	Top‐1	Top‐3	Top‐5	Top‐10
Random Baseline	77.47%	87.39%	88.88%	90.00%
Formula‐based	87.23%	92.36%	92.74%	93.06%
Model Loss‐based	87.54%	92.18%	92.56%	92.88%
**Contrastive (ours)**	**89.86%**	**92.74%**	**93.22%**	**93.52%**

The contrastive metric consistently delivers the most informative curriculum signal, achieving 89.86% top‐1 accuracy. Crucially, it outperforms the computationally massive Model Loss‐based approach (87.54%). This indicates a fundamental misalignment: a teacher model's cross‐entropy loss often reflects sequence memorization artifacts (e.g., specific token frequencies) rather than intrinsic chemical complexity. By forcing the encoder to maintain representation invariance across isomorphic SMILES views, our contrastive objective distills pure topological difficulty, providing a superior gradient pacing sequence.

#### 3.3.2 Orthogonality to Heuristics and Reaction Classes

**FIGURE 2:**
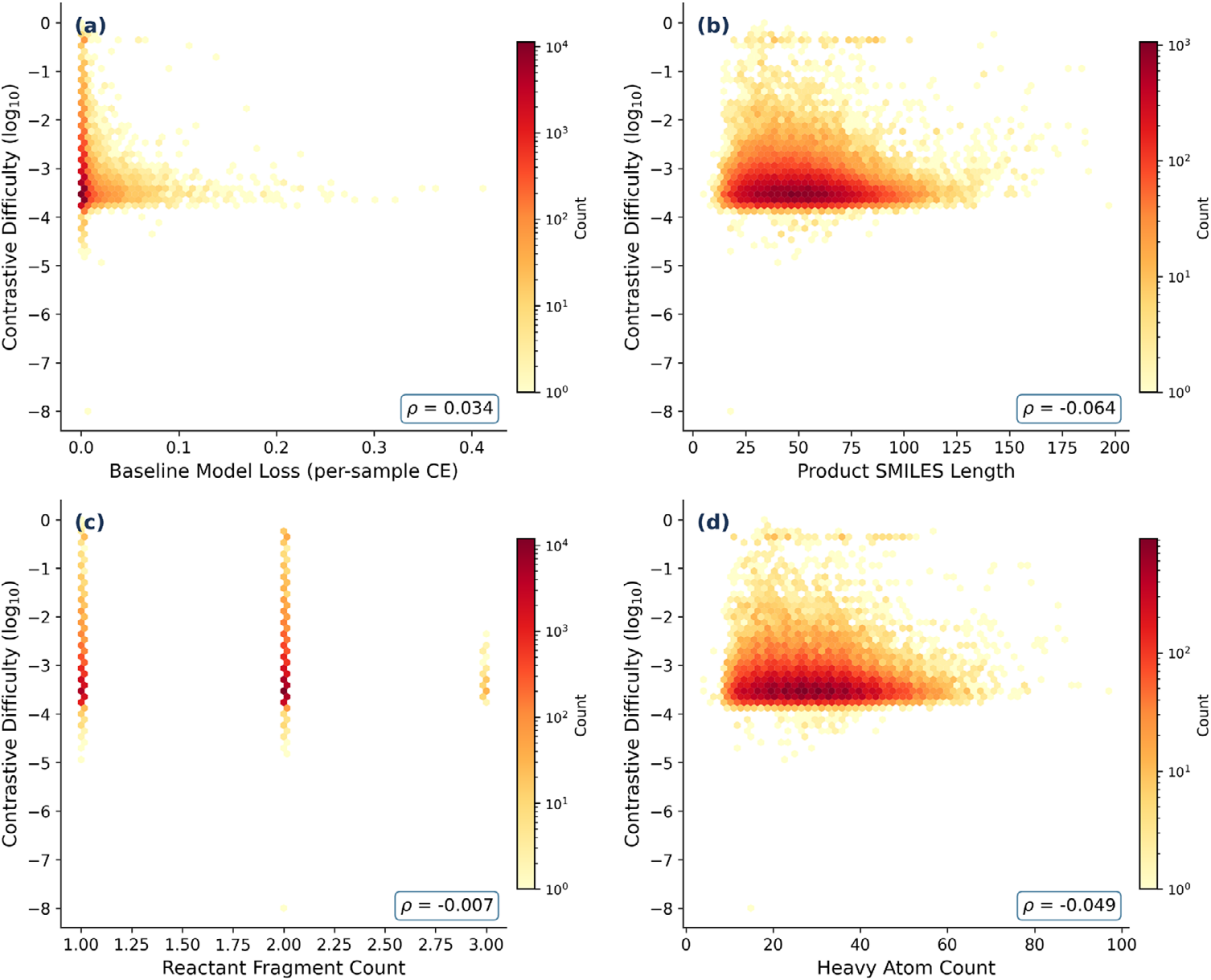
Hexbin scatter plots demonstrating what Contrastive Difficulty captures. The near‐zero Spearman correlations (ρ) confirm that our self‐supervised metric is highly orthogonal to (a) teacher model cross‐entropy loss, and static heuristics like (b) sequence length, (c) fragment count, and (d) heavy atom count.

To understand the underlying nature of contrastive difficulty, we analyze its correlation with traditional complexity proxies in Figure 2. The hexbin scatter plots reveal exceptionally weak Spearman correlation coefficients across the board: Baseline Model Loss (ρ=0.034), Product SMILES Length (ρ=−0.064), Reactant Fragment Count (ρ=−0.007), and Heavy Atom Count (ρ=−0.049). This mathematically confirms that Engine A captures an entirely orthogonal dimension of difficulty, successfully avoiding the biases of superficial 1D and 2D structural descriptors.

**FIGURE 3:**
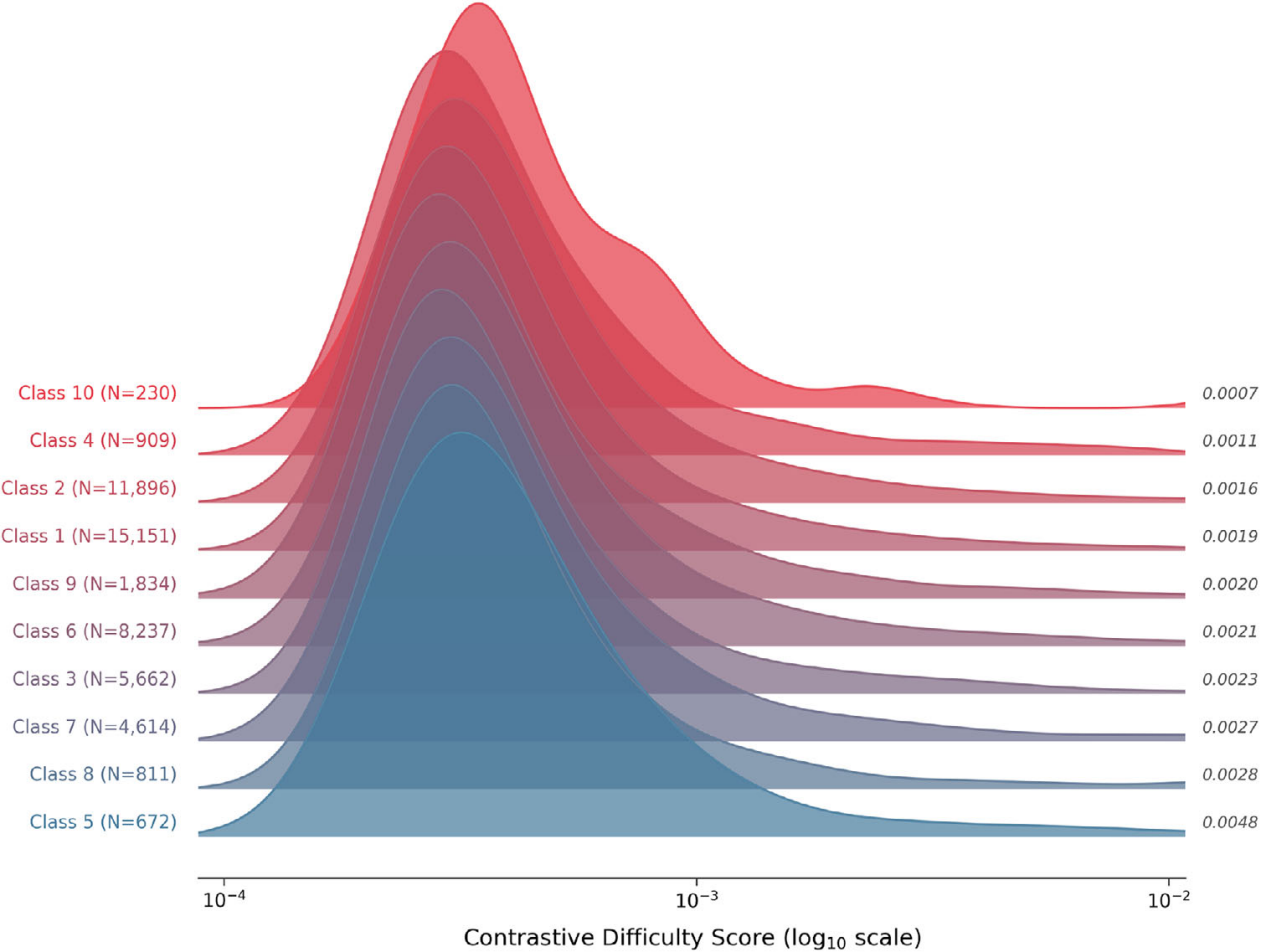
Ridgeline plot of contrastive difficulty distributions across the 10 reaction classes. The extensive overlap and broad intra‐class variance illustrate that reaction type alone is insufficient to characterize retrosynthetic complexity.

Furthermore, we analyze how this difficulty is distributed across specific chemistry types. As visualized in the Ridgeline Plot (Figure 3), there is substantial intra‐class variance. While some classes (e.g., Class 5) lean slightly harder, the distributions largely overlap. We conducted a rigorous Kruskal‐Wallis test to determine if our data‐driven difficulty merely reconstructs these coarse reaction class labels. While the test yields a statistically significant p‐value ($1.52\times 10^{-21}$) due to large sample sizes, the calculated effect size ($\varepsilon^{2}=0.0024$) is negligible. This signifies that reaction class labels explain only **0.24% of the variance** in contrastive difficulty. Thus, Engine A extracts a fine‐grained topological complexity that coarse domain classifications completely overlook.

### 3.4 Engine B Analysis: Buffering Topology‐Induced SMILES Reordering

While Engine A effectively governs pacing (the ablation of specific pacing functions is detailed in Table S1), pure curriculum learning for sequence models suffers from a critical flaw: the rapid onset of post‐curriculum overfitting once the full dataset is exposed. We posited in Section 1 that this is caused by “autoregressive myopia” and the extreme penalties inflicted by hard labels during topology‐induced SMILES string reorderings. Engine B (Label Smoothing) is introduced not as a standard regularizer, but as a topological buffer to resolve this epistemological conflict.

#### 3.4.1 Extending the Effective Learning Window

**FIGURE 4:**
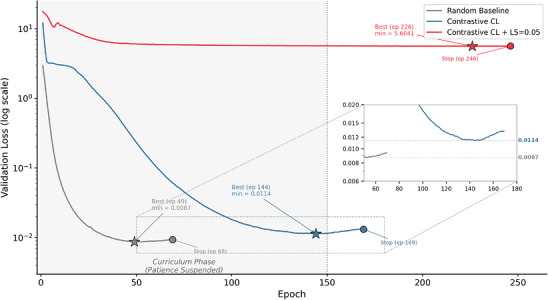
Validation loss trajectories. The Random Baseline (gray) suffers from premature convergence. Engine A Only (blue) stabilizes early training but experiences a sharp overfitting spike after the curriculum ends (epoch 150). CLRe Dual‐Engine (red) mathematically softens this topological shock, extending the effective learning window by 5×. *(Note: The absolute value of the LS loss is naturally higher due to the additive uniform distribution entropy term, but its optimization trajectory is remarkably stabilized.)*.

Figure 4 vividly illustrates the catastrophic collapse of standard optimization and the restorative power of Engine B.

The Random Baseline (gray line) is overwhelmed by early gradient noise from complex samples, prematurely converging at epoch 49 and stopping at epoch 69. Engine A (blue line) successfully stabilizes the early trajectory through linear pacing. However, the exact moment the curriculum completes (λ(t)=1 at epoch 150) and the model is exposed to the most irregular, highly complex topologies, a sharp overfitting spike occurs. The model simply cannot reconcile its local token‐prediction mechanics with the sudden demand for global, topology‐consistent structure generation dictated by hard labels.

Integrating Engine B (red line) fundamentally alters this dynamic. By allocating a concentrated, mathematically significant tolerance to non‐target tokens, Engine B cushions the penalization of string‐divergent exploration paths. The model absorbs the structural shock at epoch 150 without a validation spike, pushing the optimal convergence to epoch 226. This translates to an unprecedented **post‐curriculum learning window of over 90 epochs** (a 5× increase), allowing the model to slowly internalize the most difficult reaction mechanisms without gradient polarization.

To interpret the higher absolute loss under label smoothing, we decomposed the label‐smoothed objective (excluding padding tokens) into a hard‐label cross‐entropy term and a uniform‐distribution regularization term: (1−ε)CE(y,p)+εCE(U,p) with ε=0.05. This decomposition shows that the larger absolute LS value primarily reflects the intended additive uniform‐distribution regularization rather than degraded hard‐label optimization. Across saved CLRe LS = 0.05 validation checkpoints, the weighted uniform term contributed approximately 88.6–89.5% of the LS total, explaining why the LS curve remains numerically higher while the optimization trajectory remains stable. Full numerical validation and logged component traces are provided in the Supporting Information.

#### 3.4.2 Visualizing the Knowledge Wave

**FIGURE 5:**
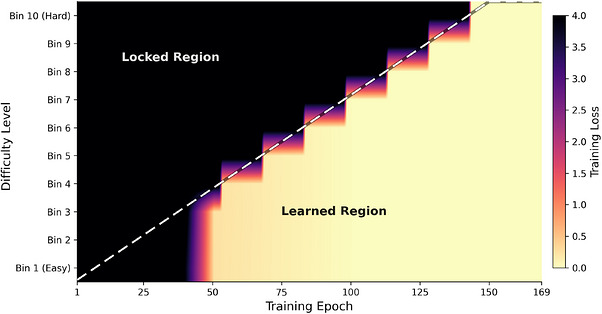
The Knowledge Wave Heatmap of the CLRe Dual‐Engine. The linear pacing boundary (λ(t), dashed white line) dynamically separates the unlocked exploration region from the locked complex region. The curriculum facilitates a smooth, wave‐like transition from high loss (dark) to low loss (light) as difficulty increases.

To vividly illustrate how CLRe systematically unlocks complex chemistry, Figure 5 presents the “Knowledge Wave” heatmap. The *y*‐axis segments the dataset into 10 difficulty bins (based on the Engine A metric).

Under standard random training, high‐difficulty bins remain perpetually “locked” because early gradient noise destroys the model's ability to learn intricate topologies. In contrast, the CLRe Dual‐Engine enforces a strict macroscopic discipline, with the linear pacing boundary (dashed white line) acting as a dynamic “knowledge frontier.”

Crucially, the heatmap reveals three profound learning dynamics:

1. **Initial Struggle and Confusion**: In the early phase (Epochs 1–50), the transition from high loss (dark) to low loss (light) for the easiest bins (Bins 1–3) spans a remarkably wide horizontal band. This visualizes the model's initial “confusion” as it painstakingly gropes to establish foundational chemical grammar and basic transformation rules.
2. **Accelerated Late‐Stage Assimilation**: As the curriculum progresses, a compounding knowledge effect emerges. The transition bands for the hardest bins (Bins 8–10) become visibly narrower. Having built a robust structural foundation from the easy data, the model no longer struggles; it can now rapidly assimilate highly complex, anomalous topologies almost immediately upon their introduction.
3. **Prevention of Complacency**: Notice that the fully “Learned Region” (light yellow) maintains a stable, non‐zero loss floor. This is the direct visual manifestation of the Label Smoothing buffer. By continuously injecting mathematical entropy, Engine B prevents the model from becoming “complacent” or overconfident in its predictions. This enforced uncertainty forces the model to maintain a state of active exploration, keeping alternative structural paths alive during beam search decoding.

### 3.5 Chemical Insights and Case Studies

**FIGURE 6:**
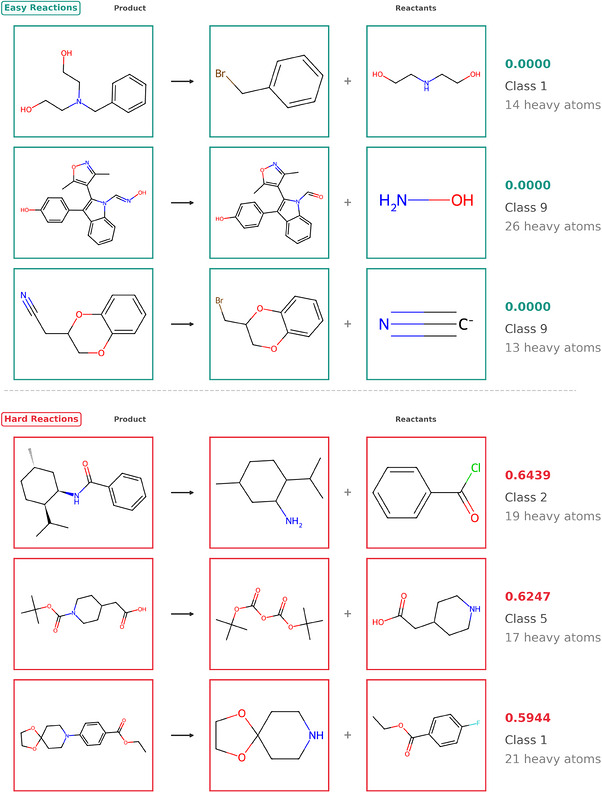
Case Study: Representative retrosynthetic transformations ranked by contrastive difficulty. **Top Row (Easy)**: Highly standardized, modular transformations with obvious synthons (e.g., simple alkylation and functional group interconversions). **Bottom Row (Hard)**: Complex topological challenges, including bulky steric hindrance (Class 2), macroscopic reagent generation such as Boc‐protection (Class 5), and intricate spiro/ketal‐like acetal systems (Class 1), which trigger high unfamiliarity.

To definitively bridge the gap between our data‐driven mathematical metric and intrinsic chemical intuition, Figure 6 visualizes reactions situated at the extremes of the contrastive difficulty spectrum.

#### 3.5.1 Validating the Familiarity Hypothesis

**Low‐Difficulty Regime (The Dense Manifold)**: Reactions generating near‐zero contrastive loss (Figure 6, Top Row) invariably involve highly standardized, localized transformations acting on ubiquitous structural motifs. These include a textbook heteroatom alkylation (Class 1) and basic functional group interconversions (Class 9). Because these localized operations dominate the high‐density regions of the USPTO data manifold, the contrastive encoder easily maps them into invariant representations. Synthetically, these targets possess obvious disconnection sites and trivial commercially available precursors, making them ideal candidates for the early‐stage curriculum warm‐up.

**High‐Difficulty Regime (The Sparse Long‐Tail)**: Conversely, reactions generating high contrastive loss (Figure 6, Bottom Row) present profound topological anomalies. The first hard example (Class 2, difficulty 0.6439) features a highly substituted, bulky aliphatic ring, demanding precise regiocontrol under severe steric hindrance. The second example (Class 5, difficulty 0.6247) represents the classic “autoregressive myopia” trap: the model must globally hallucinate a massive, independent Boc‐protection reagent from a minimal local motif. The final example (Class 1, difficulty 0.5944) involves an intricate spiro‐ketal/acetal‐like framework that drastically alters the SMILES traversal logic. These data‐driven anomalies perfectly mirror the cognitive difficulty experienced by human experts. By reserving these topologically complex samples for the late‐stage curriculum (protected by the Engine B buffer), CLRe averts early gradient polarization and successfully resolves the long‐tail of chemical complexity.

#### 3.5.2 Failure Case Analysis and Model Limitations

While the CLRe Dual‐Engine framework achieves an unprecedented 94.08% top‐1 accuracy on USPTO‐50K, analyzing the residual 5.92% error rate provides critical insights into the fundamental limitations of text‐based sequence models. A manual inspection of randomly sampled failure cases revealed three primary failure modes:

1. **Data Scarcity in the Extreme Long‐Tail (** ∼ **40%)**: The model fundamentally fails on exceptionally rare reaction classes or exotic skeletal rearrangements that appear fewer than five times in the training corpus. While macroscopic pacing (Engine A) stabilizes optimization, it cannot magically synthesize chemical rules that are statistically absent from the data manifold.
2. **Ambiguous Retrosynthetic Disconnections (** ∼ **35%)**: In highly complex natural product synthesis, a single target often possesses multiple chemically viable, orthogonal retrosynthetic routes of comparable complexity. In these instances, the sequence model's top‐1 prediction may be synthetically valid but diverges from the specific ground‐truth route documented in the USPTO database, highlighting the limitation of relying solely on exact‐match evaluation metrics.
3. **Mechanistic Opacity in Cascade Reactions (** ∼ **25%)**: For intricate domino or cascade reactions involving simultaneous, multi‐bond formations, the 1D SMILES string representation fails to capture the 3D transition state geometries and mechanistic nuances required to deduce the exact precursors.

These failure modes suggest that future breakthroughs in retrosynthesis will require hybrid architectures that fuse the linguistic capacity of CLMs with explicit 3D geometric awareness and reaction mechanism modeling, moving beyond mere string manipulation. Furthermore, while the CLRe framework improves learning efficiency on a fixed curated dataset, curriculum optimization alone cannot create examples for reaction classes or disconnection patterns that are absent or extremely rare in the training data. For such severe data scarcity, synthetic data augmentation is a complementary direction rather than a competing baseline. Future studies could combine both paradigms by using synthetic augmentation to expand rare reaction classes and then applying CLRe to organize the expanded dataset for stable and efficient assimilation of the augmented long‐tail knowledge.

## 4 Conclusion

In this work, we identified two fundamental bottlenecks restricting the fine‐tuning of Chemical Language Models for retrosynthesis: the optimization instability caused by randomly shuffled, highly complex reaction data, and the inherent mismatch between local autoregressive generation and global chemical topology. To address these challenges, we proposed **CLRe**, a synergistic dual‐engine framework.

Our first engine introduced a purely data‐driven, self‐supervised difficulty metric based on contrastive representation learning. This metric naturally quantifies the intrinsic topological complexity of molecules without relying on biased heuristics, guiding a linear curriculum strategy that stabilizes early training trajectories. Crucially, our second engine repurposed Label Smoothing as a *topological and intent buffer*. By mathematically softening the sharp gradient penalties typically inflicted during complex structural generation, this buffer effectively extended the post‐curriculum learning window by 5×. This mechanism grants the beam search algorithm the vital entropy required to explore multiple viable pathways and master the long tail of chemical complexity.

Consequently, within the controlled ReactionT5v2‐based seq2seq fine‐tuning setting studied here, CLRe achieves the strongest USPTO‐50K performance in this comparison (94.08% top‐1 accuracy, +16.61pp) and systematically mitigates performance disparities across different reaction classes, with robust scalability validated on the massive USPTO‐MIT corpus.

Despite these advances, our framework has certain limitations. First, while our contrastive difficulty metric effectively captures 2D topological complexity, it currently lacks explicit representations of 3D stereochemical features, which are critical for asymmetric synthesis. Second, although Label Smoothing serves as an effective heuristic buffer, it uniformly penalizes sequence divergence without explicitly distinguishing between fundamentally invalid chemical structures and valid, alternative synthetic pathways. While CLRe significantly improves predictive performance, a key limitation of current retrosynthesis benchmarks such as USPTO‐50K is the presence of potential one‐pot/cascade‐like reactions without procedure‐level annotations. Such cases are mechanistically opaque and should not be interpreted as clean single‐step disconnections. Future work may benefit from explicit curation or classification steps that separate these boundary cases from standard single‐step reactions.

Future work will aim to incorporate 3D geometric priors into the contrastive metric and develop chemically‐aware smoothing loss functions. Ultimately, by seamlessly bridging self‐supervised macroscopic pacing with microscopic topological buffering, this work provides a rigorous optimization paradigm that successfully aligns data‐driven learning dynamics with intrinsic chemical intuition.

## Conflicts of Interest

The authors declare no conflicts of interest.

## Supporting information


**Supporting File 1**: advs76827‐sup‐0001‐SuppMat.pdf.

**Supporting File 2**: advs76827‐sup‐0002‐DataSet.zip.

## Data Availability

The data that support the findings of this study are available in GitHub (wengong‐jin/nips17‐rexgen) at https://github.com/wengong‐jin/nips17‐rexgen/tree/master/USPTO. These data were derived from the following resources available in the public domain:‐ GitHub (wengong‐jin/nips17‐rexgen), https://github.com/wengong‐jin/nips17‐rexgen/tree/master/USPTO
