# Supplementary material for: CLRe: A Synergistic Dual‐Engine Framework for One‐Step Retrosynthesis Prediction
Source: Adv Sci (Weinh). 2026 Jul 29:e76827. Online ahead of print. doi: 10.1002/advs.76827 (PMC13418513; doi:10.1002/advs.76827)
Supplement: Supplementary file 1 — Supporting File 1: advs76827‐sup‐0001‐SuppMat.pdf. [file ADVS-9999-e76827-s001.pdf]

Supporting Information

CLRe: A Synergistic Dual-Engine Framework for One-Step Retrosynthesis Prediction

Tianhao Su*[1], Xitao Wang*[1], Musen Li*[1], Guanhua Qin[2], Shunbo Hu†[1,3,4], and Tong-Yi Zhang†[1]

[1] *Material Genome Institute, Institute for the Conservation of Cultural Heritage, Institute for Quantum Science and Technology, Shanghai University, Shanghai 200444, China*

[2] *Shanghai Xin Chang Rong Semiconductor Materials Co., Ltd., Shanghai 201900, China*

[3] *Key Laboratory of Silicate Cultural Heritage Conservation (Shanghai University), Ministry of Education, Shanghai 200444, China*

[4] *Institute for the Conservation of Cultural Heritage, Key Laboratory of Silicate Cultural Relics Conservation (Ministry of Education), Shanghai University, Shanghai 200444, China*

S1. Pacing Function Dynamics

Table S1 details the ablation of various curriculum pacing functions on USPTO-50K (without label smoothing). Linear pacing provides the most robust monotonic expansion of the structural manifold, preventing the early overwhelming representation collapse seen in logarithmic pacing and avoiding the topological starvation characteristic of exponential pacing.

Table S1: Ablation of pacing functions on USPTO-50K using contrastive difficulty (without label smoothing).

**Pacing Function**	**Top-1**	**Top-3**	**Top-5**	**Top-10**	**Best Epoch**
**Linear**	**89.86%**	**92.74%**	**93.22%**	**93.52%**	**144**
Logarithmic	88.92%	92.48%	92.96%	93.28%	138
Exponential	88.54%	92.22%	92.68%	93.02%	142
Step	89.72%	92.68%	93.16%	93.46%	146
Cosine	89.34%	92.56%	93.04%	93.34%	140

*These authors contributed equally to this work.

†Corresponding author.

Table S2: Per-class top-1 accuracy (%) on the USPTO-50K test set.

Class	Random Baseline	Formula CL	Engine A Only	CLRe (Dual-Engine)
1 (Heteroatom alkylation)	82.26	94.52	93.48	94.19
2 (Acylation)	86.51	95.31	95.48	96.30
3 (C-N bond formation)	64.77	80.18	80.37	91.19
4 (C-C bond formation)	72.09	83.72	88.37	96.51
5 (Protection)	40.00	52.86	58.57	94.29
6 (Deprotection)	68.65	83.61	83.35	92.77
7 (Reduction)	75.62	91.74	91.53	93.80
8 (Oxidation)	72.04	87.10	84.95	93.55
9 (Functional group interconv.)	72.84	92.59	87.65	91.98
10 (Other/Unclassified)	76.19	90.48	95.24	95.24
**Overall**	**77.47**	**87.23**	**89.86**	**94.08**
**Inter-class gap (max–min)**	**46.51**	**42.45**	**36.91**	**5.32**

S2. The Imperative of Pretrained Chemical Knowledge

Figure S1 shows that applying the curriculum to a randomly initialized network fails catastrophically, yielding a top-1 accuracy of only 3.92%. Large-scale pretraining supplies the chemical grammar, while CLRe provides the optimized path for fine-tuning that knowledge on difficult retrosynthetic reasoning tasks.

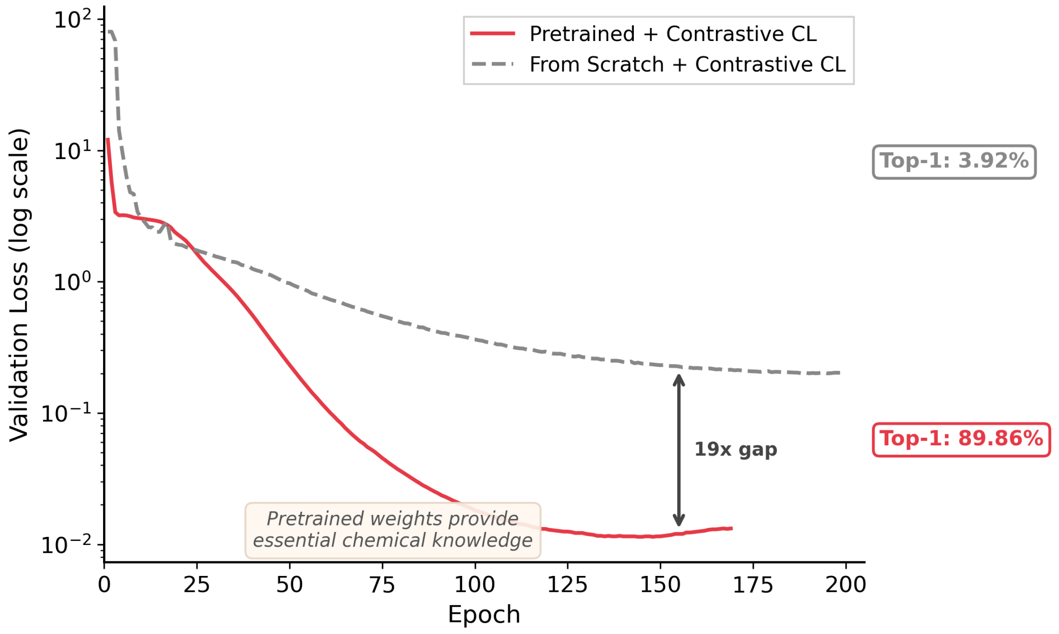

Figure S1: Validation loss comparison between pretrained and from-scratch initialization under the identical CLRe framework.

S3. Post-hoc Round-Trip Validation Protocol and Results

To complement canonical exact-match top-k accuracy on the single-reference USPTO-50K benchmark, we performed a post-hoc round-trip validation analysis using saved retrosynthesis predictions. A candidate is counted as relaxed-correct if it either exactly matches the reference reactants or is independently validated to regenerate the target product.

We used two validators. First, we used the public learned forward model `sagawa/ReactionT5v2-forward` as a learned verifier. Second, we built a deterministic train-only template-based validator from atom-mapped USPTO-50K training reactions, explicitly excluding reactions from the test sets. Templates were extracted with RDChiral, reversed into forward SMARTS, and executed with RDKit reaction APIs.

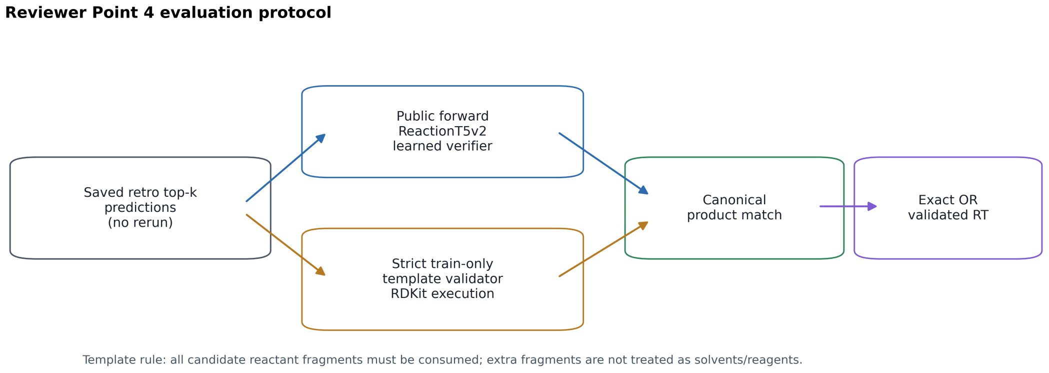

Figure S2: Round-trip evaluation protocol used for supplementary analysis.

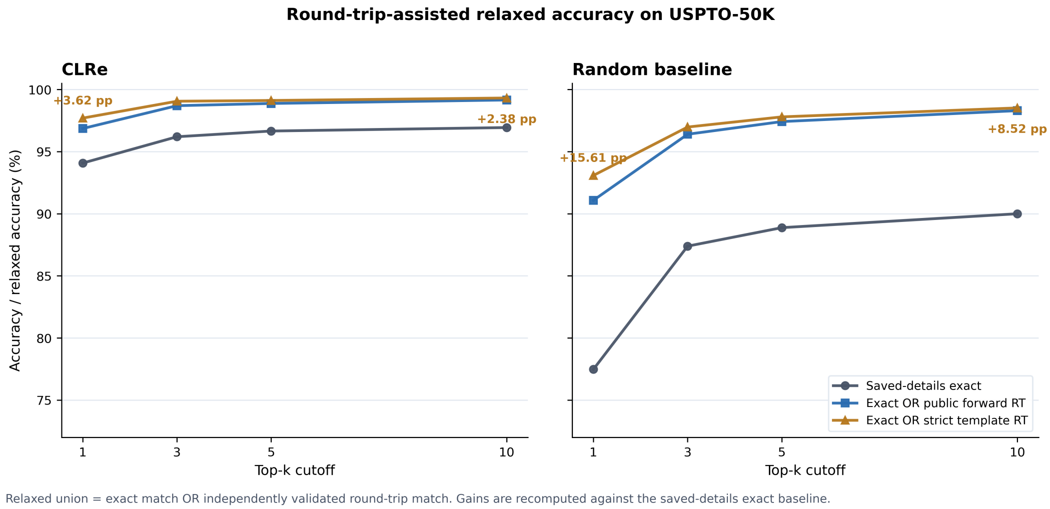

Figure S3: Main round-trip validation results for CLRe and the random baseline.

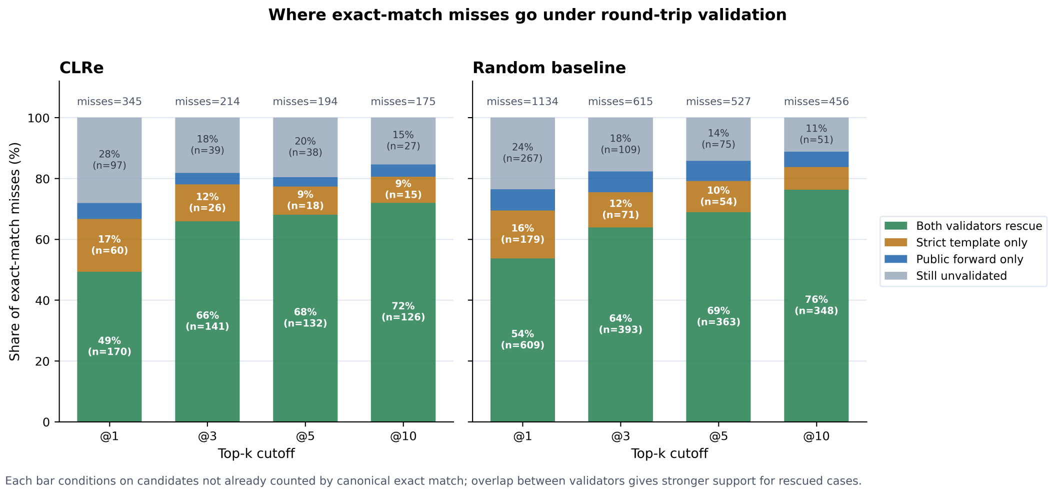

Figure S4: Overlap between learned and template-based validators for CLRe exact-match misses.

Table S3: Complete relaxed-accuracy results.

Model	Top-k	Saved-details exact	Public union	Public gain	Template union	Template gain
CLRe	@1	94.08%	96.86%	2.78 pp	97.70%	3.62 pp
CLRe	@3	96.20%	98.70%	2.50 pp	99.06%	2.86 pp
CLRe	@5	96.66%	98.88%	2.22 pp	99.12%	2.46 pp
CLRe	@10	96.94%	99.16%	2.22 pp	99.32%	2.38 pp
Random baseline	@1	77.47%	91.08%	13.61 pp	93.08%	15.61 pp
Random baseline	@3	87.39%	96.40%	9.01 pp	96.98%	9.59 pp
Random baseline	@5	88.88%	97.42%	8.54 pp	97.80%	8.92 pp
Random baseline	@10	90.00%	98.30%	8.30 pp	98.52%	8.52 pp

Table S4: CLRe exact-miss decomposition.

Top-k	Exact misses	Both validators rescue	Template only	Public only	Still unvalidated
@1	345	170 (49.3%)	60 (17.4%)	18 (5.2%)	97 (28.1%)
@3	214	141 (65.9%)	26 (12.1%)	8 (3.7%)	39 (18.2%)
@5	194	132 (68.0%)	18 (9.3%)	6 (3.1%)	38 (19.6%)
@10	175	126 (72.0%)	15 (8.6%)	7 (4.0%)	27 (15.4%)

S4. Hardest-5% Reaction Analysis

To test whether CLRe improves genuinely difficult reactions, we ranked all USPTO-50K test reactions by the CLRe contrastive difficulty score and selected the globally hardest 5% subset, yielding 251 hard test cases out of 5,002 test reactions.

Table S5: Per-class distribution of the globally hardest 5% USPTO-50K test reactions.

Class	Test n	Hard n	Hard % within class	Hard subset composition %	Mean difficulty (hard)
1	1550	65	4.19%	25.90%	0.0458
2	1216	69	5.67%	27.49%	0.0158
3	545	23	4.22%	9.16%	0.0497
4	86	5	5.81%	1.99%	0.0268
5	70	3	4.29%	1.20%	0.0108
6	775	43	5.55%	17.13%	0.0327
7	484	30	6.20%	11.95%	0.0322
8	93	2	2.15%	0.80%	0.0089
9	162	10	6.17%	3.98%	0.0571
10	21	1	4.76%	0.40%	0.0022

Table S6: Strict-protocol top-k accuracy on the hardest 5% test reactions.

Method	Top-1	Top-3	Top-5	Top-10	Validity	n
Random Baseline	77.69%	89.64%	90.84%	90.84%	98.80%	251
CLRe LS = 0.05	93.63%	96.41%	96.81%	96.81%	98.41%	251

Table S7: Absolute improvement on the hardest 5% subset under the strict protocol.

Metric	Random Baseline	CLRe LS = 0.05	Absolute improvement
Top-1	77.69%	93.63%	+15.94 pp
Top-3	89.64%	96.41%	+6.77 pp
Top-5	90.84%	96.81%	+5.98 pp
Top-10	90.84%	96.81%	+5.98 pp

Table S8: Sensitivity under a relaxed canonical-match protocol.

Protocol	Method	Top-1	Top-3	Top-5	Top-10	n
beam=10, exact string match	Random Baseline	77.69%	89.64%	90.84%	90.84%	251
beam=10, exact string match	CLRe LS = 0.05	93.63%	96.41%	96.81%	96.81%	251
beam=20, canonical match	Random Baseline	80.48%	90.44%	92.43%	92.43%	251
beam=20, canonical match	CLRe LS = 0.05	94.42%	97.61%	97.61%	97.61%	251

S5. Label-Smoothing Objective Decomposition

To clarify the optimization behavior under label smoothing, we decomposed the PyTorch label-smoothed cross-entropy into

$$\mathcal{L}_{\text{LS}} = (1-\varepsilon)\,\text{CE}(y,p) + \varepsilon\,\text{CE}(U,p),$$

with $\varepsilon = 0.05$ and padding tokens excluded.

Using saved CLRe LS = 0.05 checkpoints, we performed a post-hoc fixed-probe decomposition on the same USPTO-50K validation probe. The manual decomposition matched `torch.nn.CrossEntropyLoss(label_smoothing=0.05,ignore_index=pad_id)` with a maximum absolute difference of 1.49×10^{-7}. The weighted uniform-distribution term contributed approximately 88.6–89.5% of the total LS objective across the saved trajectory.

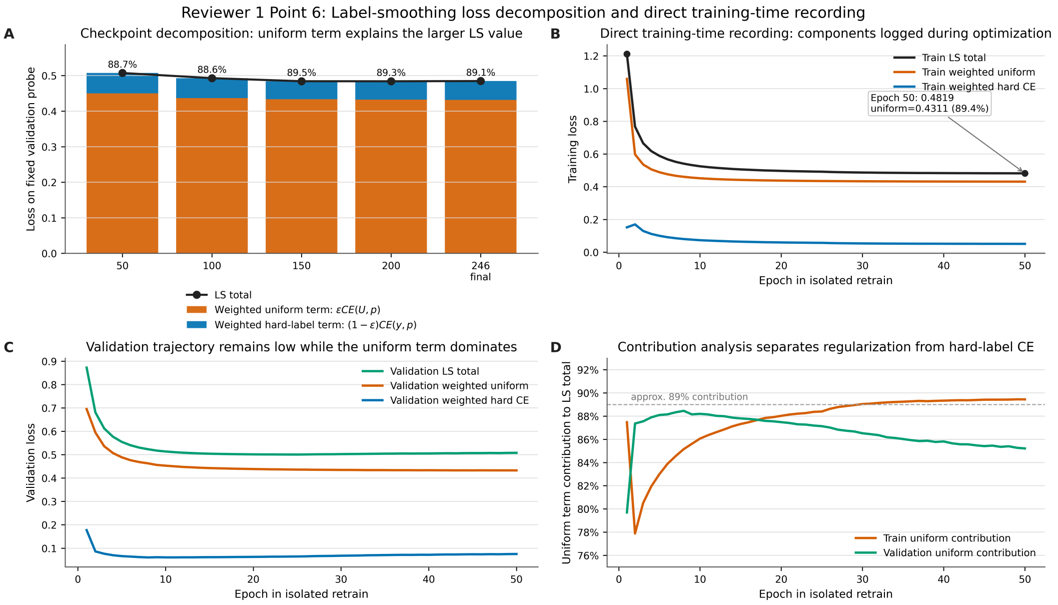

Figure S5: Checkpoint-level decomposition of the CLRe LS objective.

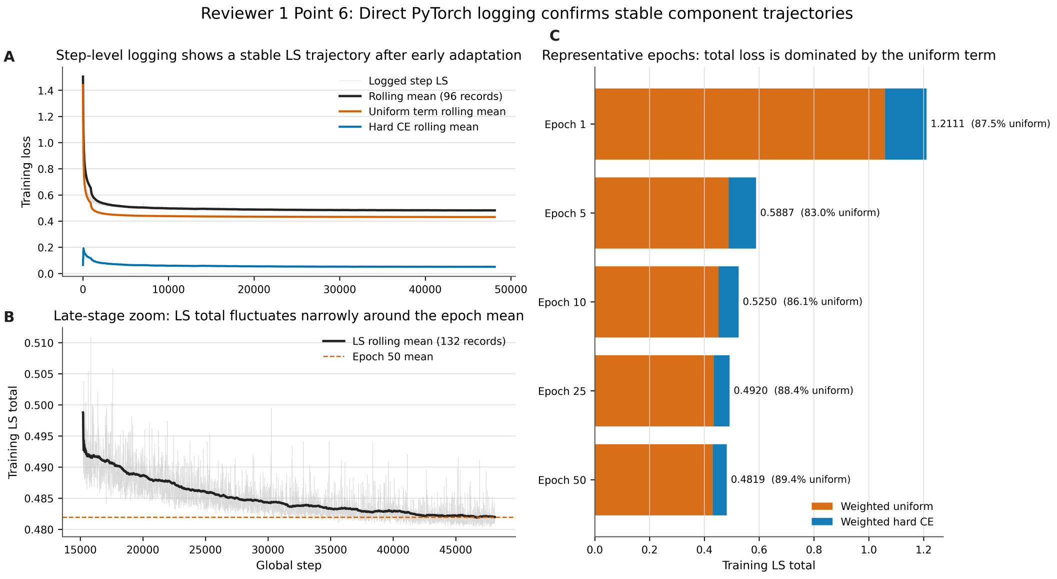

Figure S6: Training-time stability of the isolated 50-epoch CLRe LS retraining run.

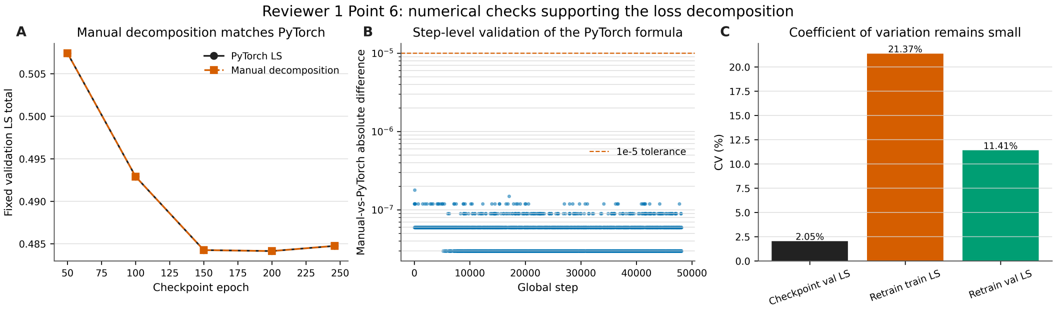

Figure S7: Numerical validation of the manual LS decomposition against the PyTorch objective.

Second, we ran a short isolated 50-epoch CLRe LS = 0.05 retraining experiment and directly logged both loss components during optimization. At epoch 50, the training LS total was 0.4819, consisting of 0.0509 weighted hard-label CE and 0.4311 weighted uniform loss. The directly logged manual decomposition matched PyTorch `CrossEntropyLoss(label_smoothing=0.05)` throughout training, with a maximum step-level difference of 1.79×10^{-7}.

S6. Controlled Grokking Audit of Random Baseline and CLRe Extension

To test whether the random-order baseline was merely undertrained rather than fundamentally limited, we conducted a controlled extended-training audit under a common 300-epoch upper budget. We report a four-checkpoint comparison under a unified evaluation: the original random best checkpoint from the early-stop run, the random no-early-stop epoch-300 checkpoint, the CLRe LS = 0.05 source best checkpoint (epoch 226), and the CLRe LS = 0.05 post-best extension checkpoint (epoch 300).

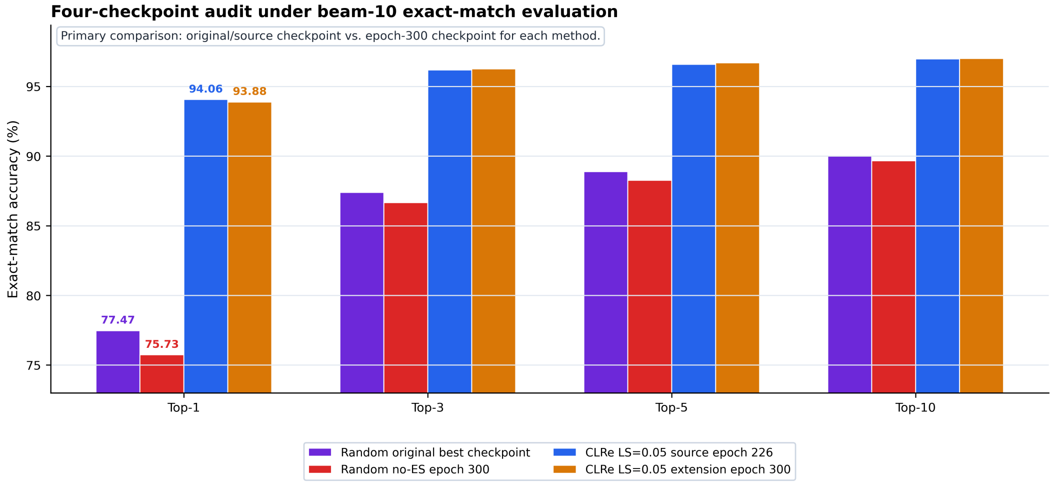

Figure S8: Four-checkpoint Top-k audit under common evaluation.

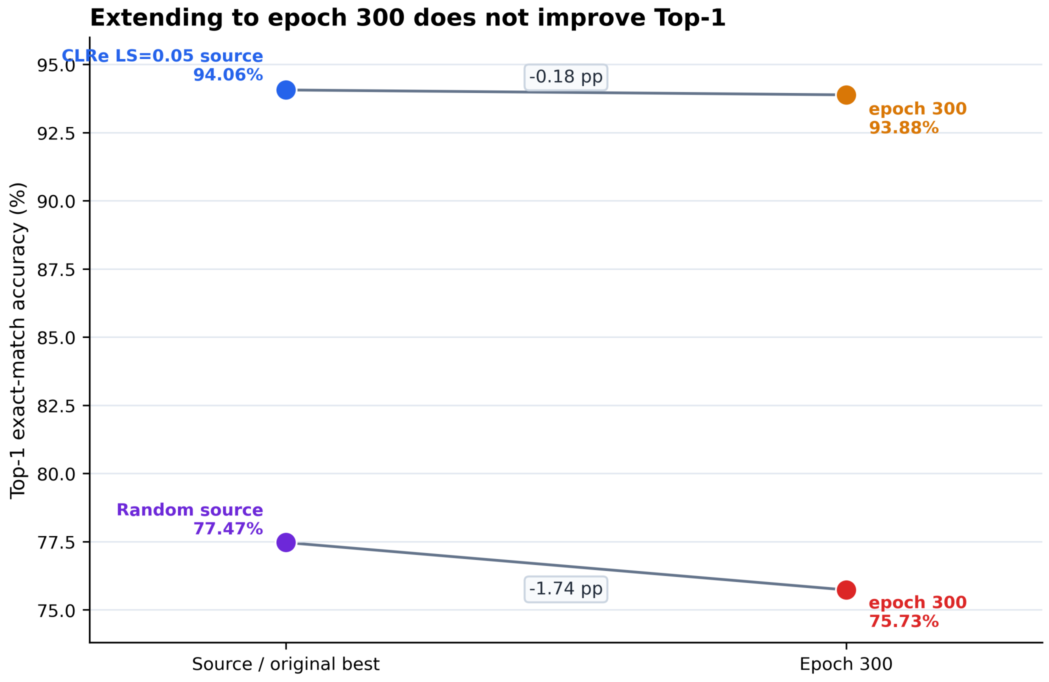

Figure S9: Paired Top-1 change from source/original-best to epoch 300.

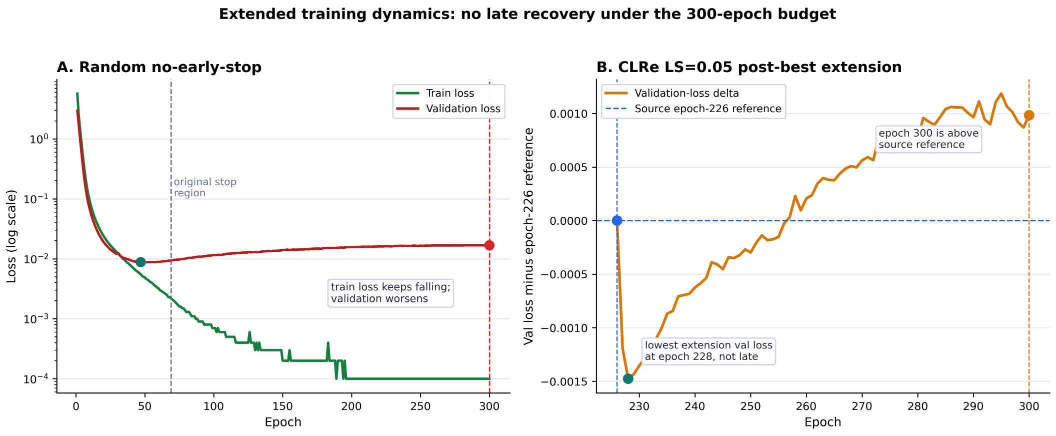

Figure S10: Extended-training dynamics for Random and CLRe LS = 0.05.

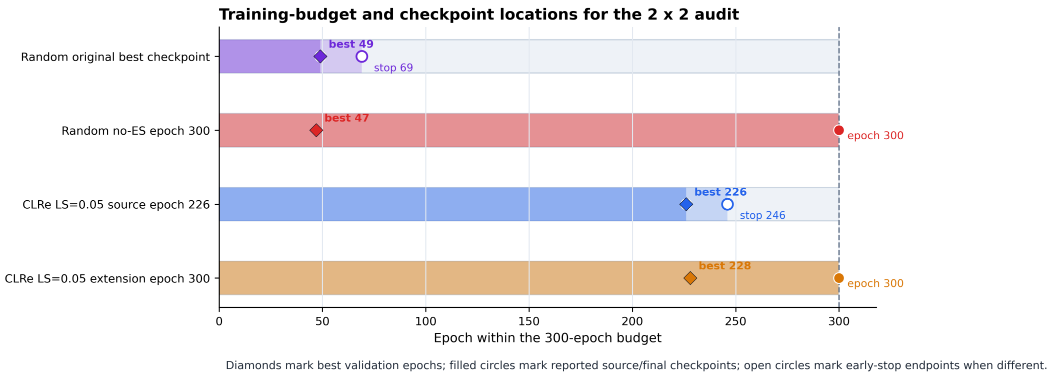

Figure S11: Epoch-budget and checkpoint-location summary.

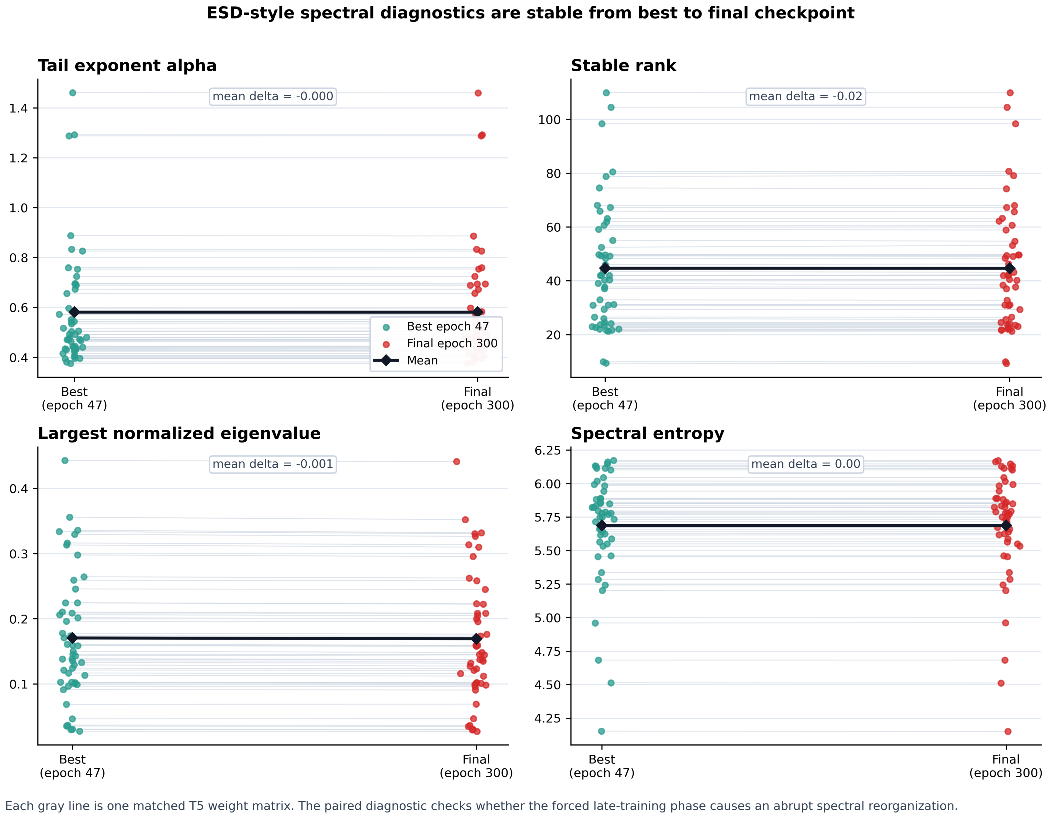

Figure S12: Paired WeightWatcher/ESD-style spectral diagnostics for the random no-early-stop run.

The no-early-stop random baseline reached its best validation loss at epoch 47 (0.0088). After the original stopping region around epoch 69, validation loss did not recover: the best post-epoch-69 value was 0.0095 at epoch 70, whereas the epoch-300 validation loss increased to 0.0168 while training loss decreased to 0.0001. On the test set, the best checkpoint achieved Top-1/3/5/10 accuracies of 77.77/87.60/89.14/90.06%, while the final epoch-300 checkpoint dropped to 75.73/86.67/88.26/89.66%.

The spectral analysis was similarly stable rather than phase-transition-like: the mean fitted power-law tail exponent α changed only from 0.581103 to 0.580962, with mean absolute paired delta 0.000409 and maximum absolute paired delta 0.001770. These results do not support a late Grokking-style recovery within the tested 300-epoch budget.

S7. CLRe Data-Exposure Milestones Under Linear Pacing

Under the linear pacing schedule, the active data fraction follows $\lambda(t) = \min(t/(0.5 \times 300), 1)$, so the active subset expands cumulatively from the easiest prefix to the full training set and reaches 100% exposure at epoch 150.

Table S9: CLRe data-exposure milestones under linear curriculum pacing.

Epoch	Active training samples	Active data fraction	Interpretation
1	270 / 40,512	0.7%	Curriculum starts from the easiest prefix.
50	13,504 / 40,512	33.3%	Easy and intermediate examples are jointly trained.
100	27,008 / 40,512	66.7%	Harder examples are progressively added, while earlier examples remain included.
144	38,891 / 40,512	96.0%	Late curriculum-expansion stage; most training data are already active.
150	40,512 / 40,512	100.0%	Full training set is active from this point onward.

S8. Dummy Visible-Complexity Baseline

We introduced a simplified visible-complexity baseline based solely on the smallest product fragment, scored as the sum of its heavy atoms and rings. This transparent heuristic provides a strong control for testing whether complex hand-designed difficulty formulas are truly necessary.

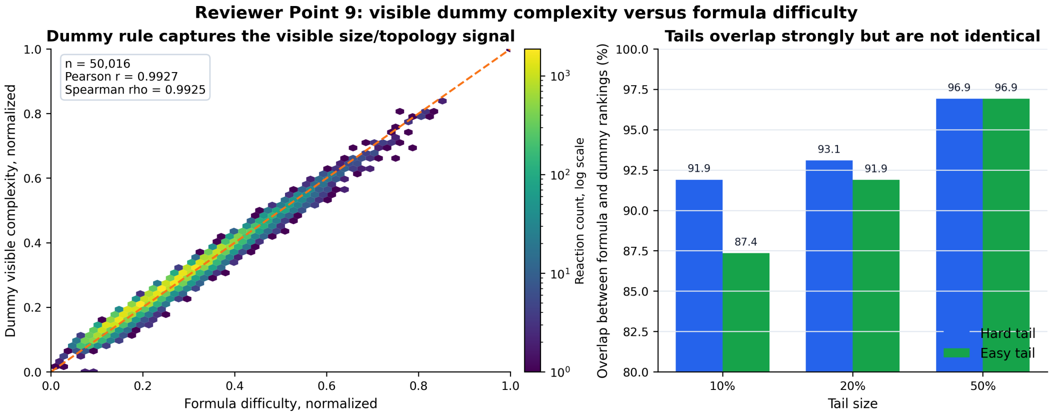

Figure S13: Relationship between the original formula-based difficulty score and the reviewer-inspired dummy visible-complexity score.

Table S10 follows the main-text USPTO-50K evaluation protocol used for the Formula CL baseline, with beam = 10 and exact SMILES matching. Tables S11 and S12 report the correlation and tail-overlap diagnostics between the formula score and the dummy visible-complexity score.

Table S10: Main-text protocol comparison.

Method	Top-1	Top-3	Top-5	Top-10	Validity	n
Formula CL based on [45]	87.23%	92.36%	92.74%	93.06%	99.72%	5002
Dummy complexity CL	90.16%	92.36%	92.64%	92.84%	99.78%	5002

Table S11: Difficulty-score relationship.

Comparison	Pearson r	Spearman ρ	n
Formula difficulty vs. dummy visible complexity	0.9927	0.9925	50,016

Table S12: Tail overlap between formula and dummy rankings.

Tail size	Hard-tail overlap	Easy-tail overlap
10%	91.90%	87.36%
20%	93.10%	91.89%
50%	96.92%	96.92%
