# Supplementary figures and images for: CLRe: A Synergistic Dual‐Engine Framework for One‐Step Retrosynthesis Prediction
Source: Adv Sci (Weinh). 2026 Jul 29:e76827. Online ahead of print. doi: 10.1002/advs.76827 (PMC13418513; doi:10.1002/advs.76827)

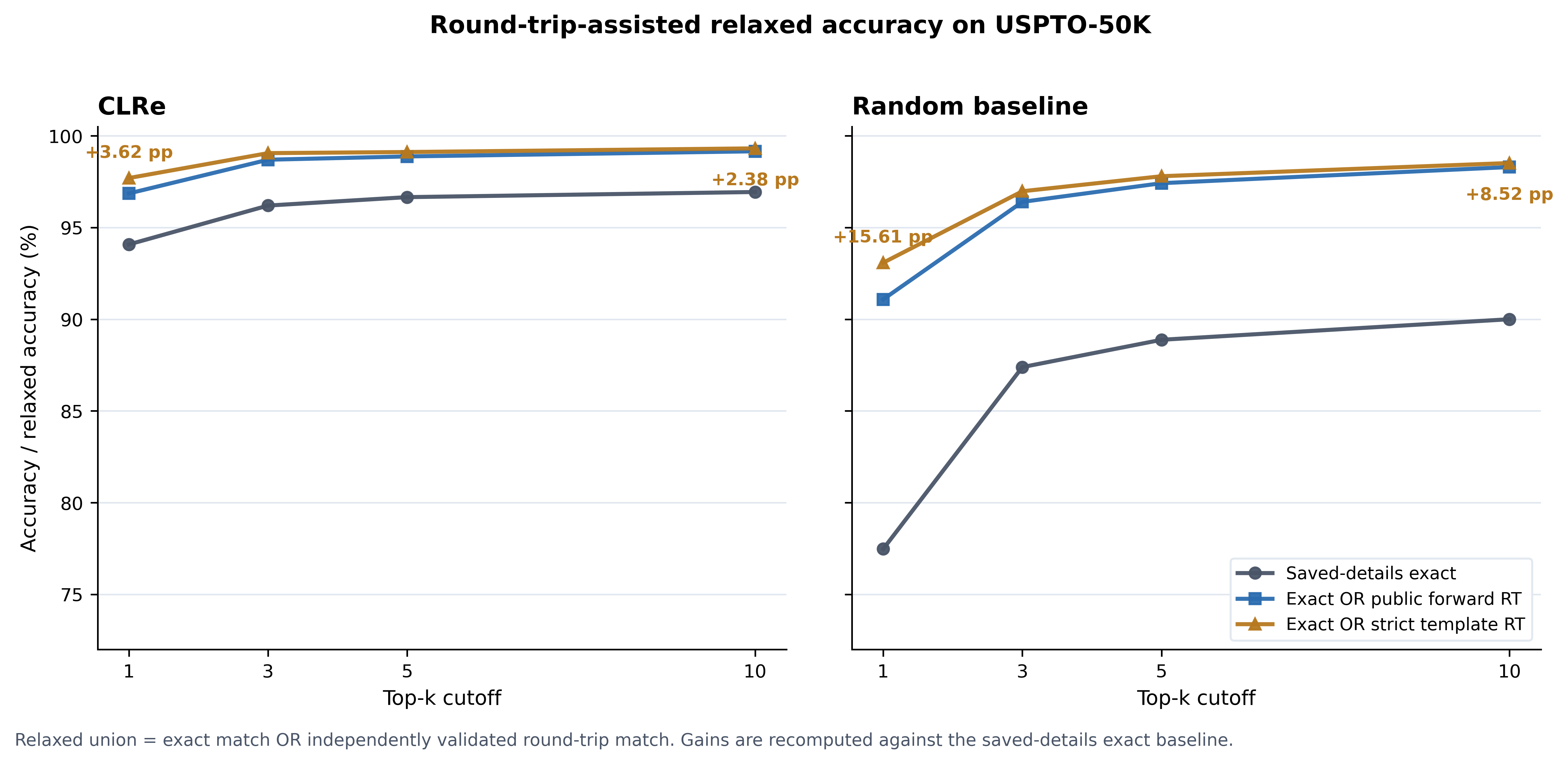

Supplement: Supplementary file 2 — Supporting File 2: advs76827‐sup‐0002‐DataSet.zip. [file ADVS-9999-e76827-s002.zip › outputs/reviewer_point4_template_roundtrip/figures/figure_point4_main_accuracy.png]

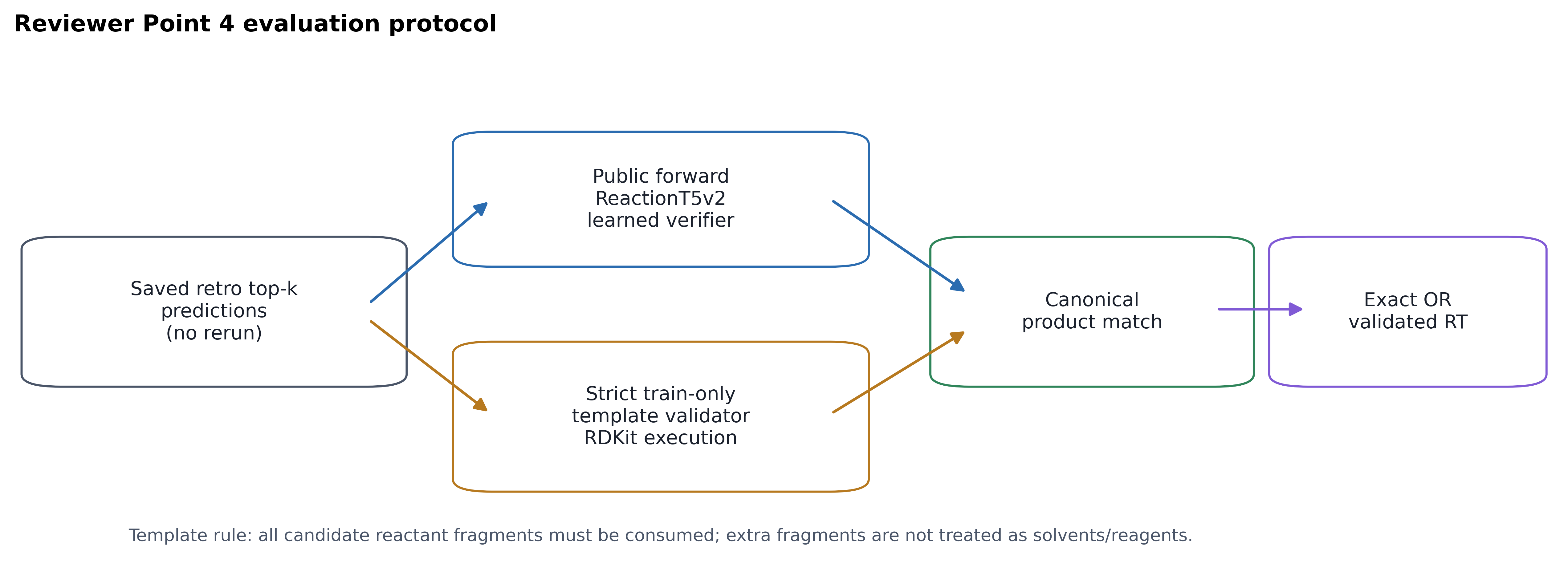

Supplement: Supplementary file 2 — Supporting File 2: advs76827‐sup‐0002‐DataSet.zip. [file ADVS-9999-e76827-s002.zip › outputs/reviewer_point4_template_roundtrip/figures/figure_point4_protocol.png]

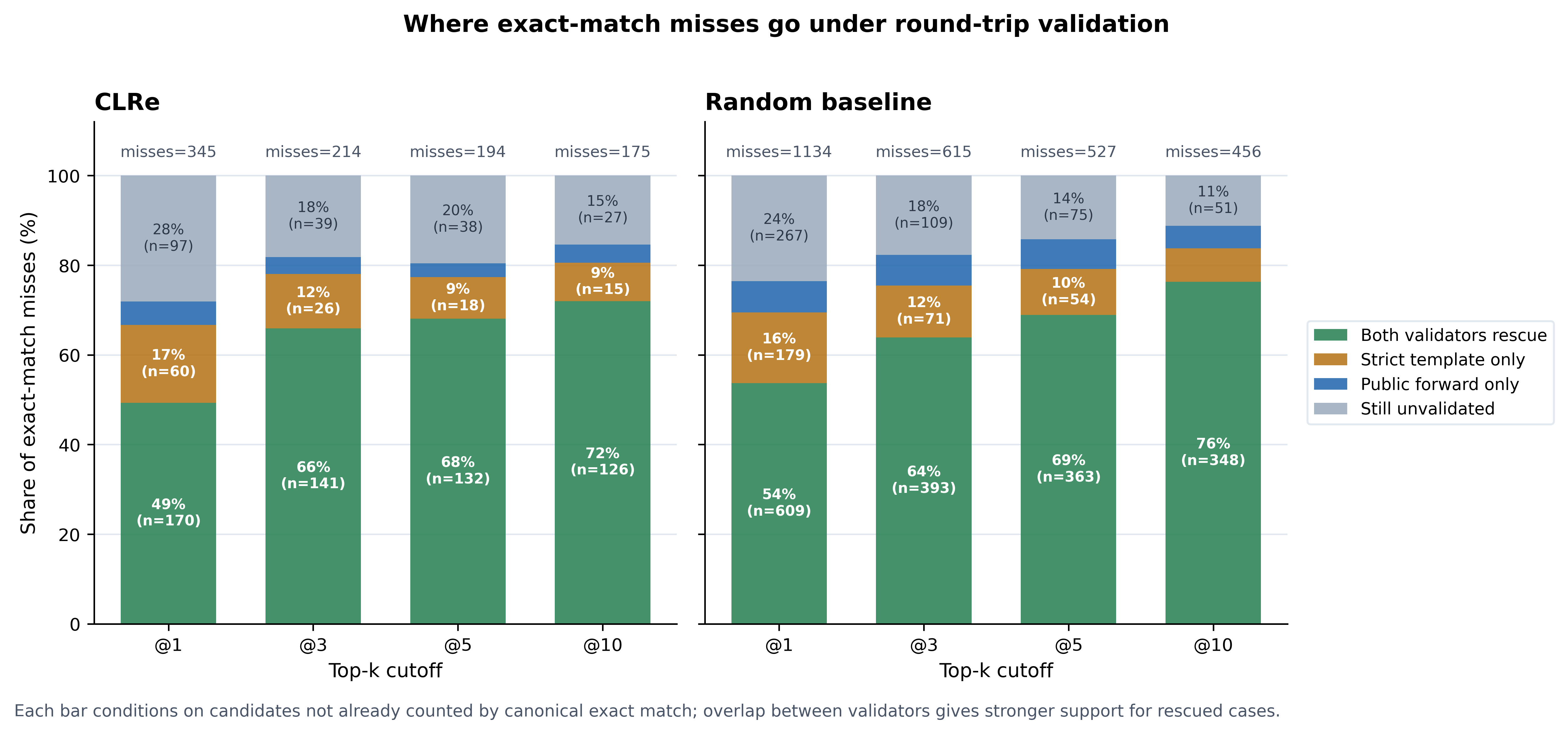

Supplement: Supplementary file 2 — Supporting File 2: advs76827‐sup‐0002‐DataSet.zip. [file ADVS-9999-e76827-s002.zip › outputs/reviewer_point4_template_roundtrip/figures/figure_point4_validator_overlap.png]

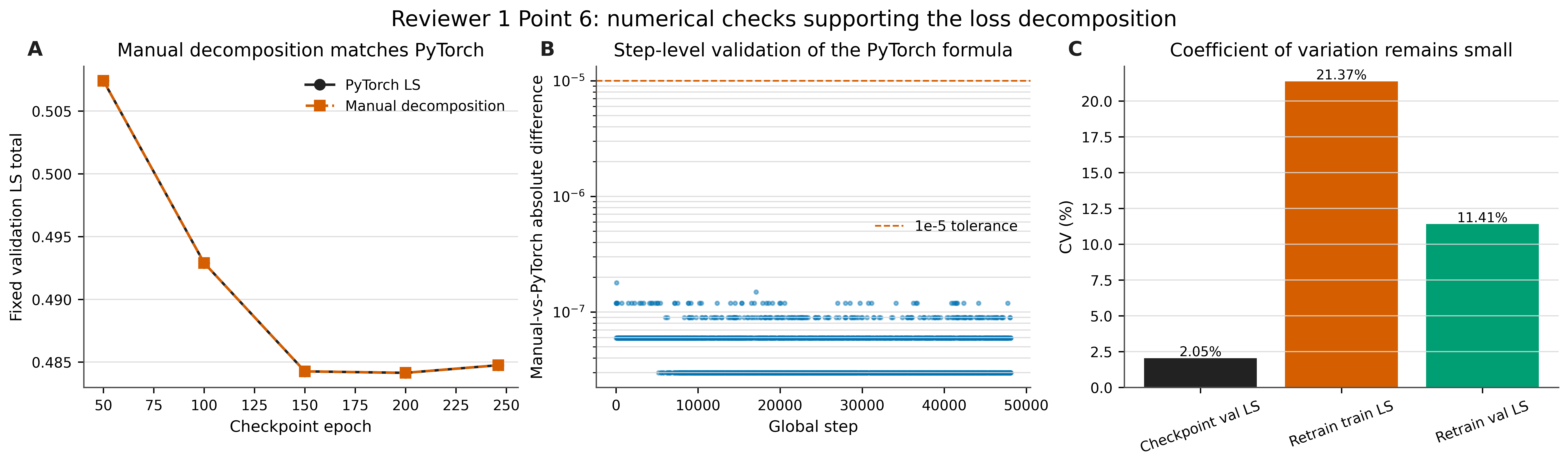

Supplement: Supplementary file 2 — Supporting File 2: advs76827‐sup‐0002‐DataSet.zip. [file ADVS-9999-e76827-s002.zip › outputs/reviewer_point6_publication_figures/figure_point6_formula_validation.png]

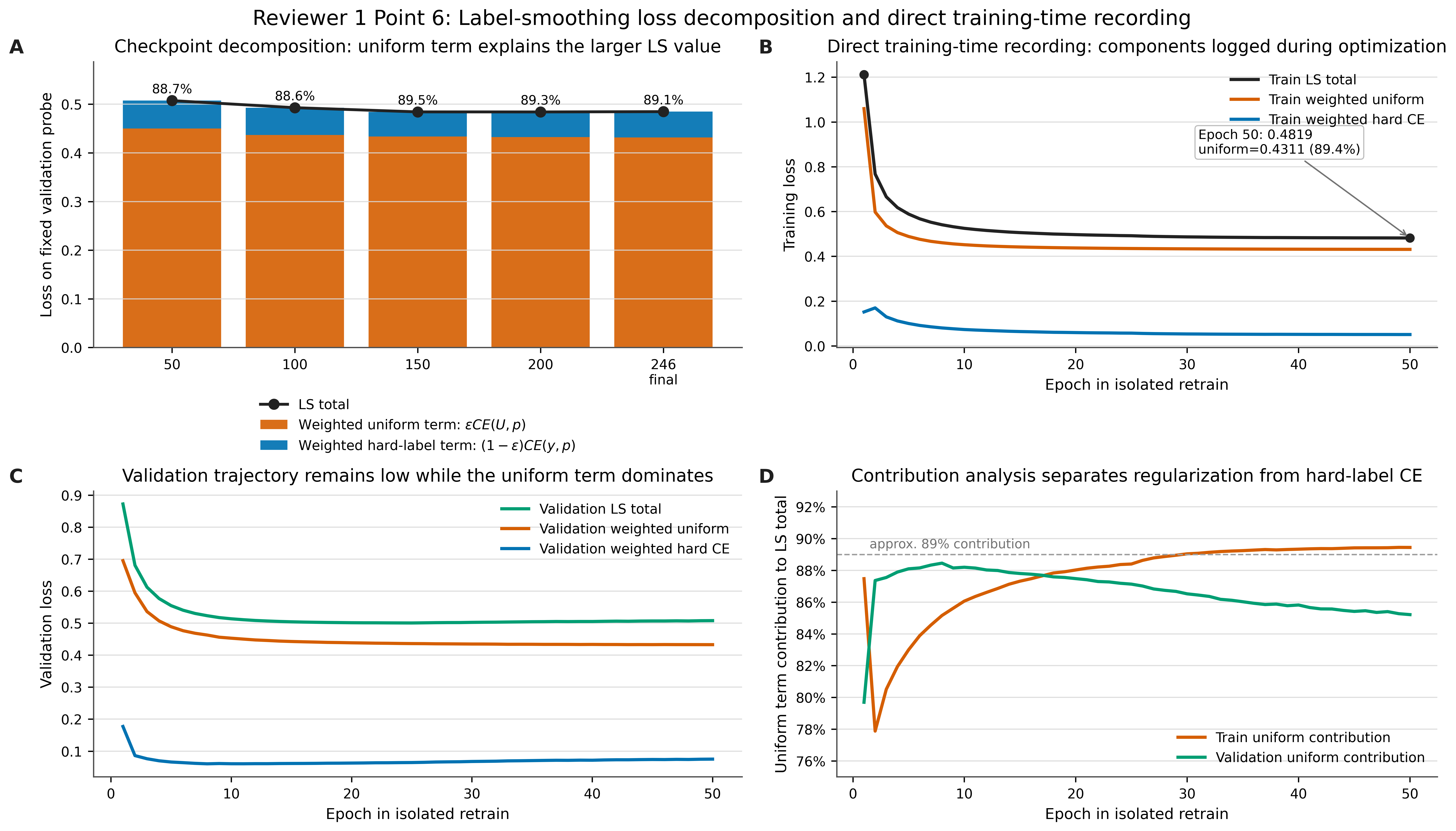

Supplement: Supplementary file 2 — Supporting File 2: advs76827‐sup‐0002‐DataSet.zip. [file ADVS-9999-e76827-s002.zip › outputs/reviewer_point6_publication_figures/figure_point6_loss_decomposition_overview.png]

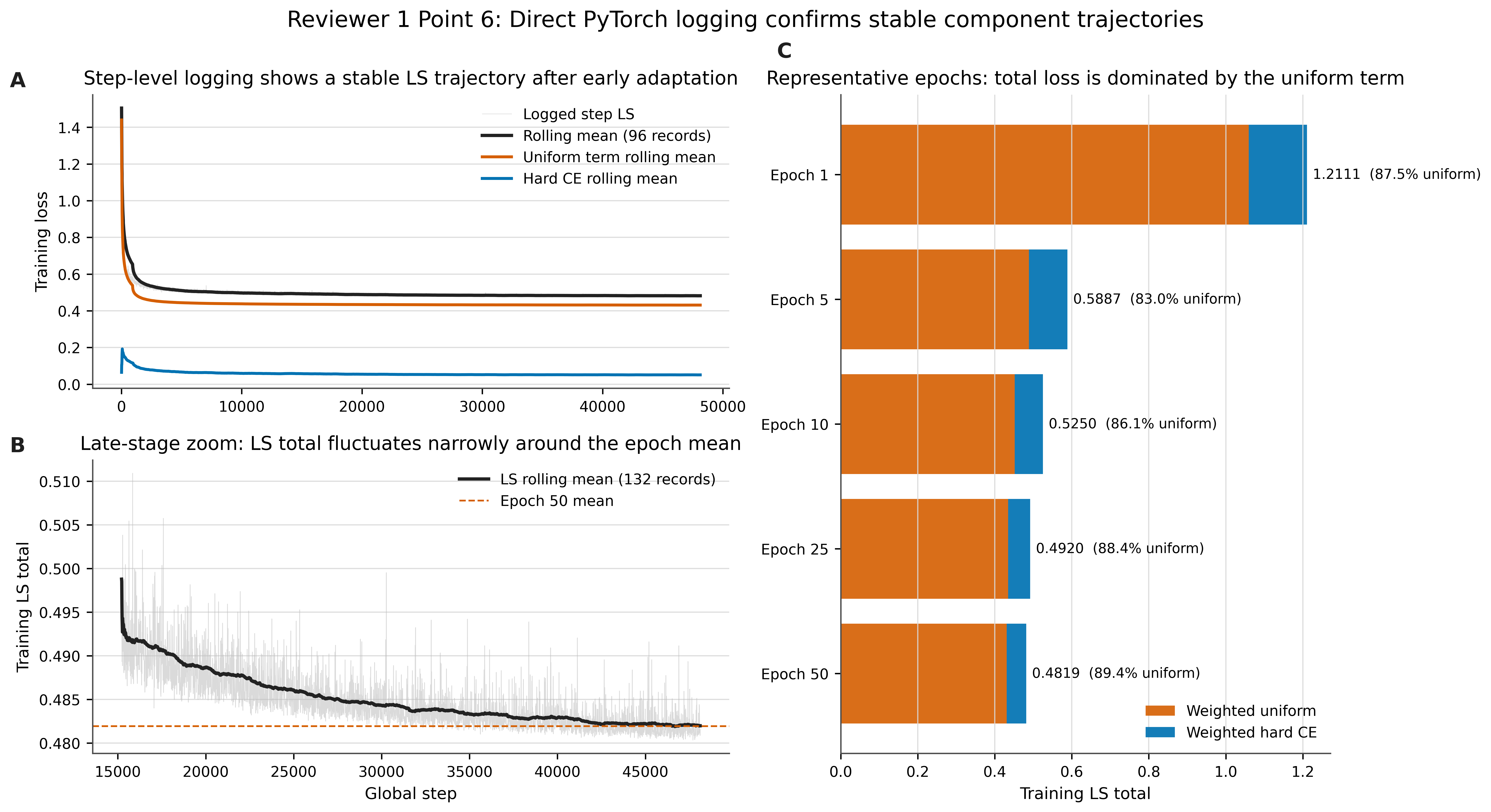

Supplement: Supplementary file 2 — Supporting File 2: advs76827‐sup‐0002‐DataSet.zip. [file ADVS-9999-e76827-s002.zip › outputs/reviewer_point6_publication_figures/figure_point6_training_time_stability.png]

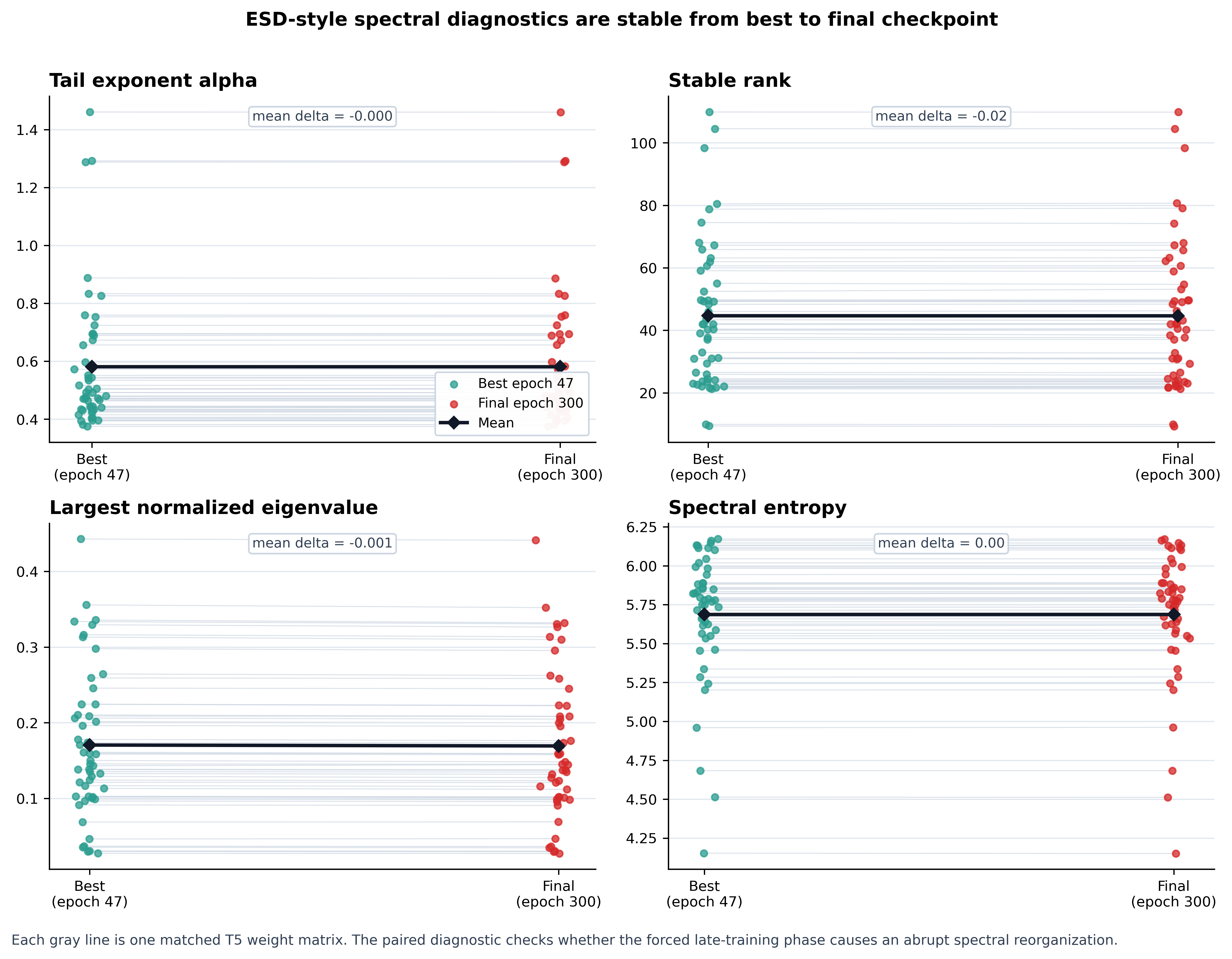

Supplement: Supplementary file 2 — Supporting File 2: advs76827‐sup‐0002‐DataSet.zip. [file ADVS-9999-e76827-s002.zip › outputs/reviewer_point7_grokking_esd/figures/figure_point7_esd_stability.png]

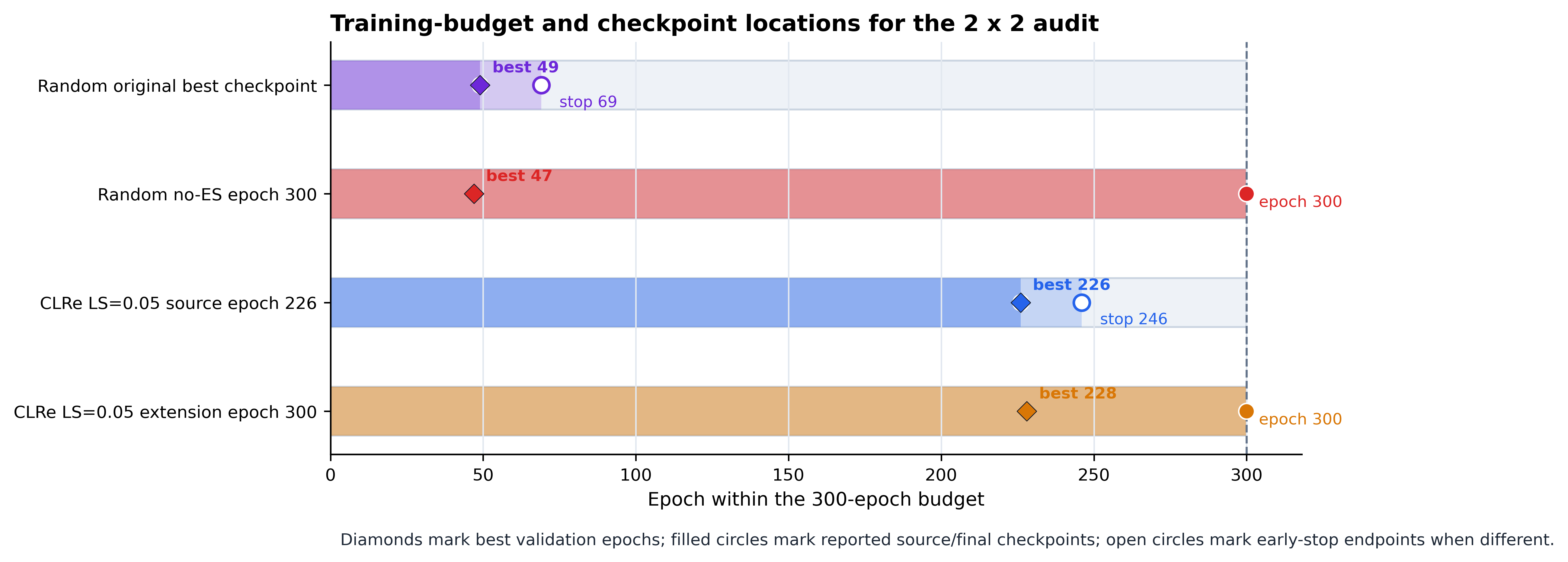

Supplement: Supplementary file 2 — Supporting File 2: advs76827‐sup‐0002‐DataSet.zip. [file ADVS-9999-e76827-s002.zip › outputs/reviewer_point7_grokking_esd/figures/figure_point7_v2_budget_summary.png]

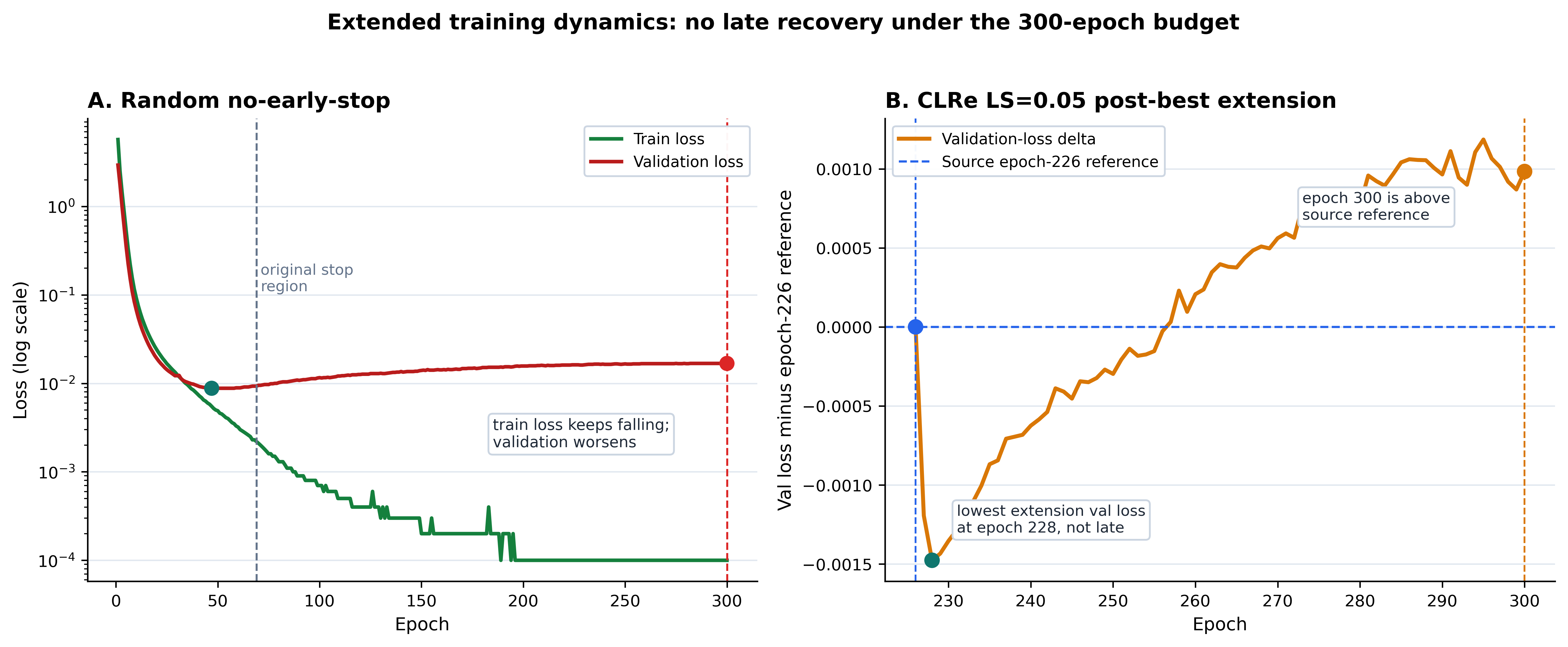

Supplement: Supplementary file 2 — Supporting File 2: advs76827‐sup‐0002‐DataSet.zip. [file ADVS-9999-e76827-s002.zip › outputs/reviewer_point7_grokking_esd/figures/figure_point7_v2_extension_dynamics.png]

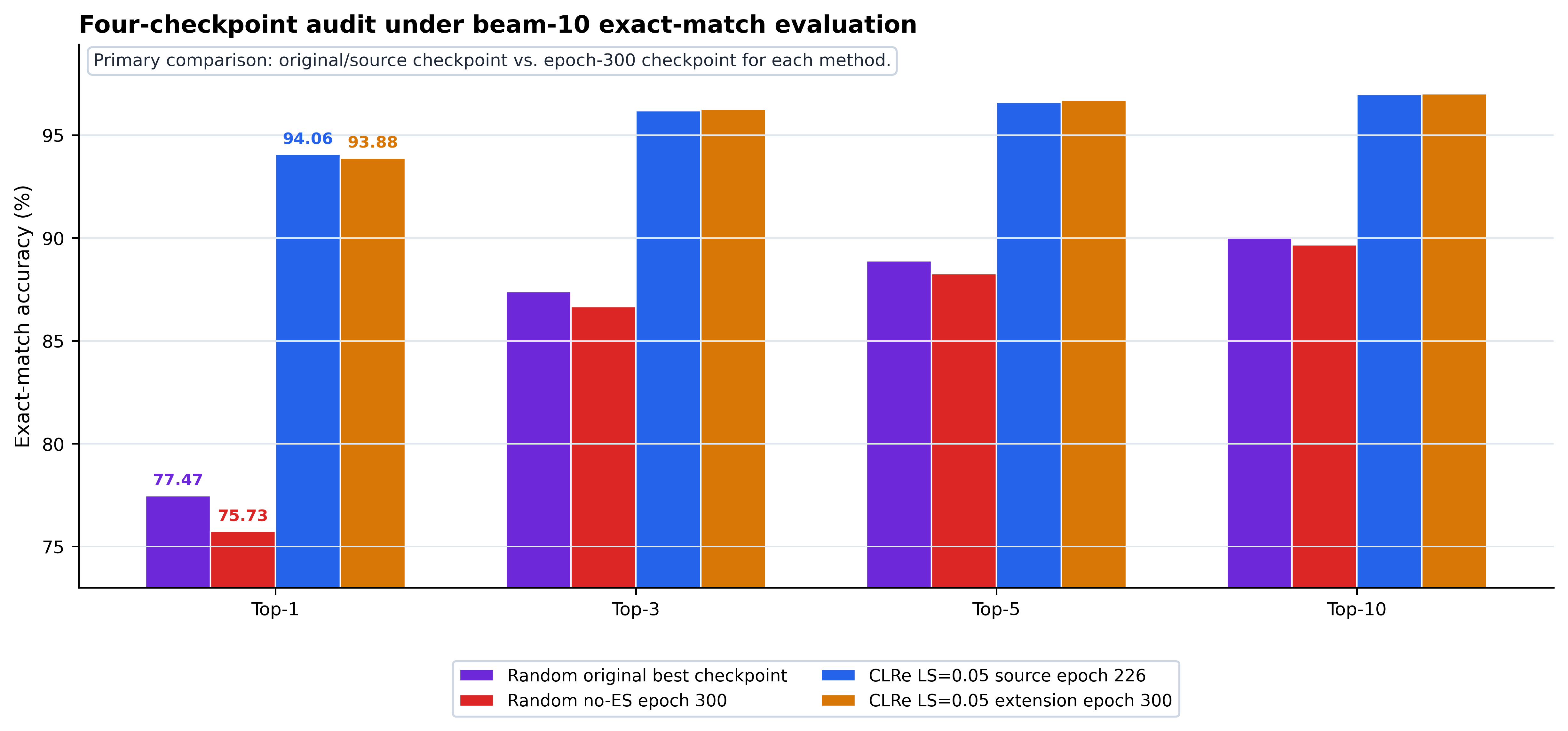

Supplement: Supplementary file 2 — Supporting File 2: advs76827‐sup‐0002‐DataSet.zip. [file ADVS-9999-e76827-s002.zip › outputs/reviewer_point7_grokking_esd/figures/figure_point7_v2_four_checkpoint_topk.png]

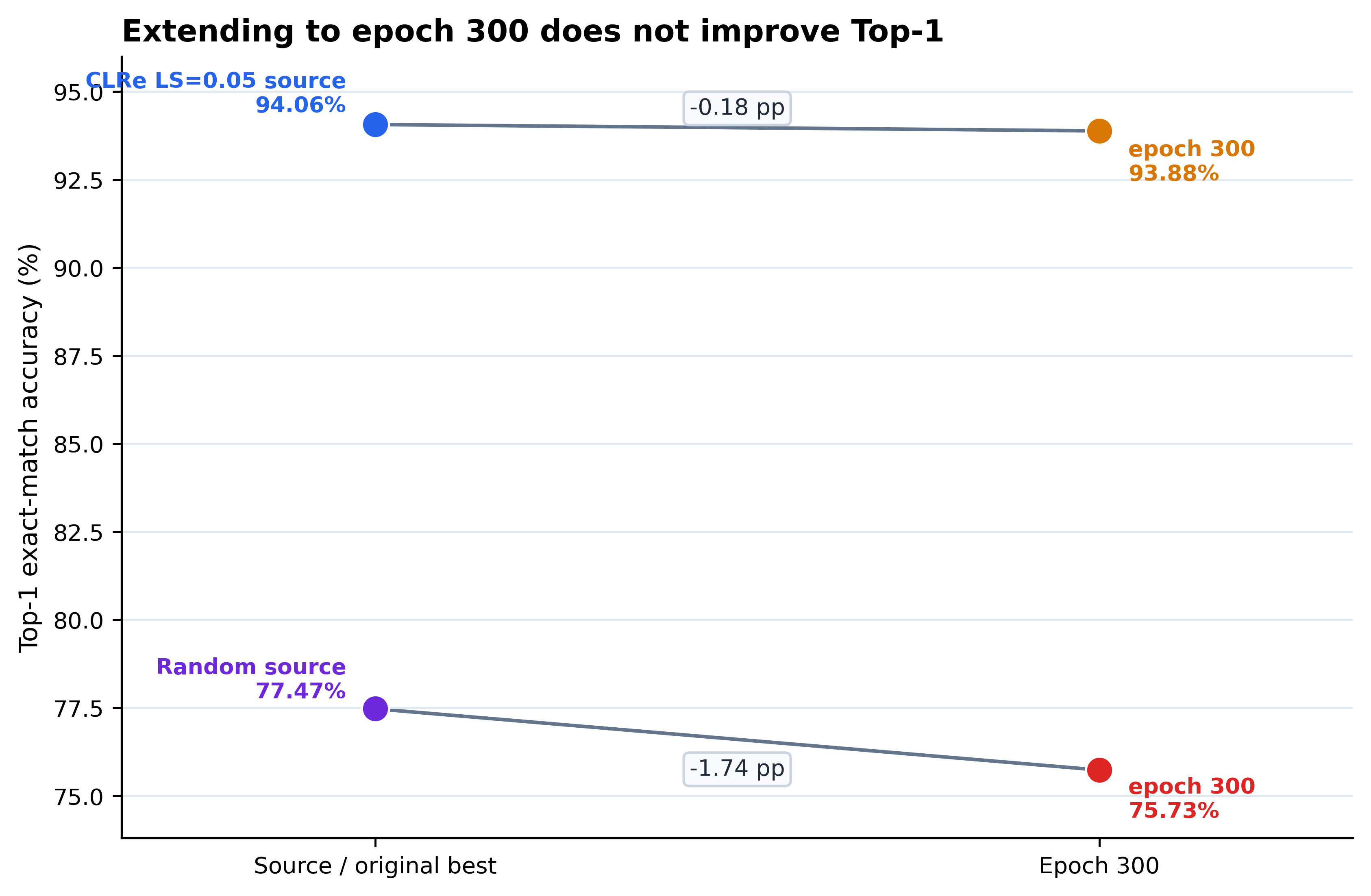

Supplement: Supplementary file 2 — Supporting File 2: advs76827‐sup‐0002‐DataSet.zip. [file ADVS-9999-e76827-s002.zip › outputs/reviewer_point7_grokking_esd/figures/figure_point7_v2_top1_delta.png]

Point 7: layer-family mean delta alpha (final - best)

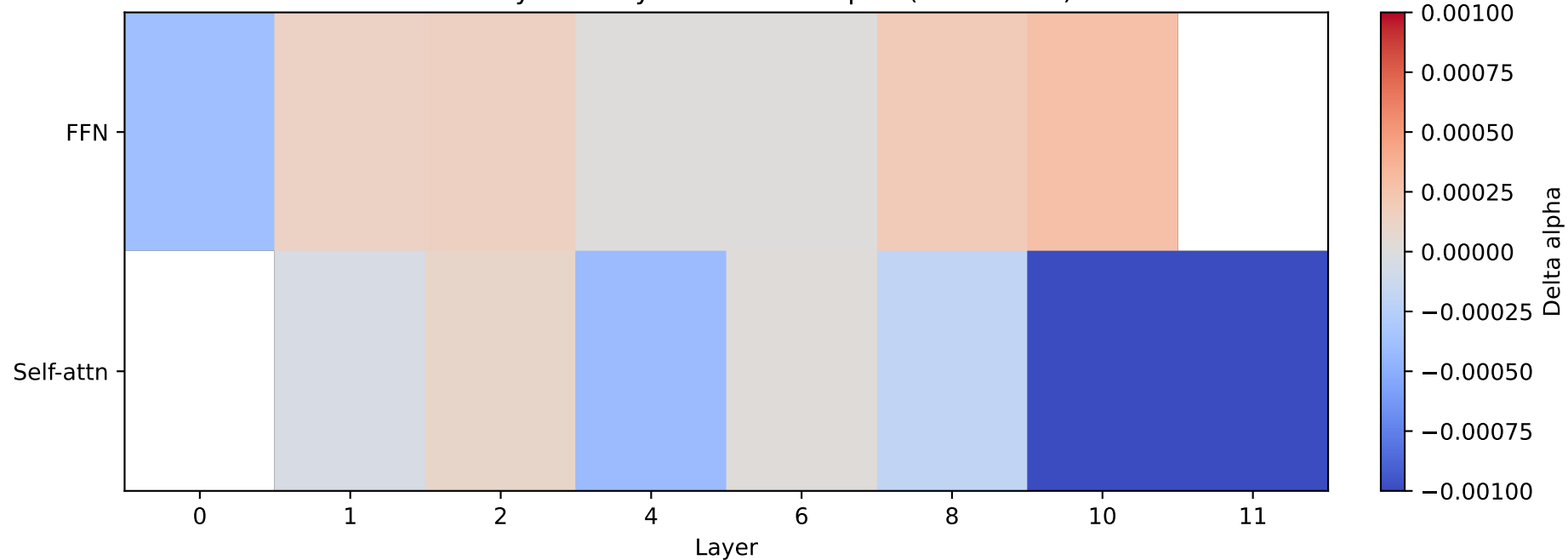

Supplement: Supplementary file 2 — Supporting File 2: advs76827‐sup‐0002‐DataSet.zip. [file ADVS-9999-e76827-s002.zip › outputs/reviewer_point7_grokking_esd/supplementary_point7/point7_supp_esd_delta_heatmap.pdf]

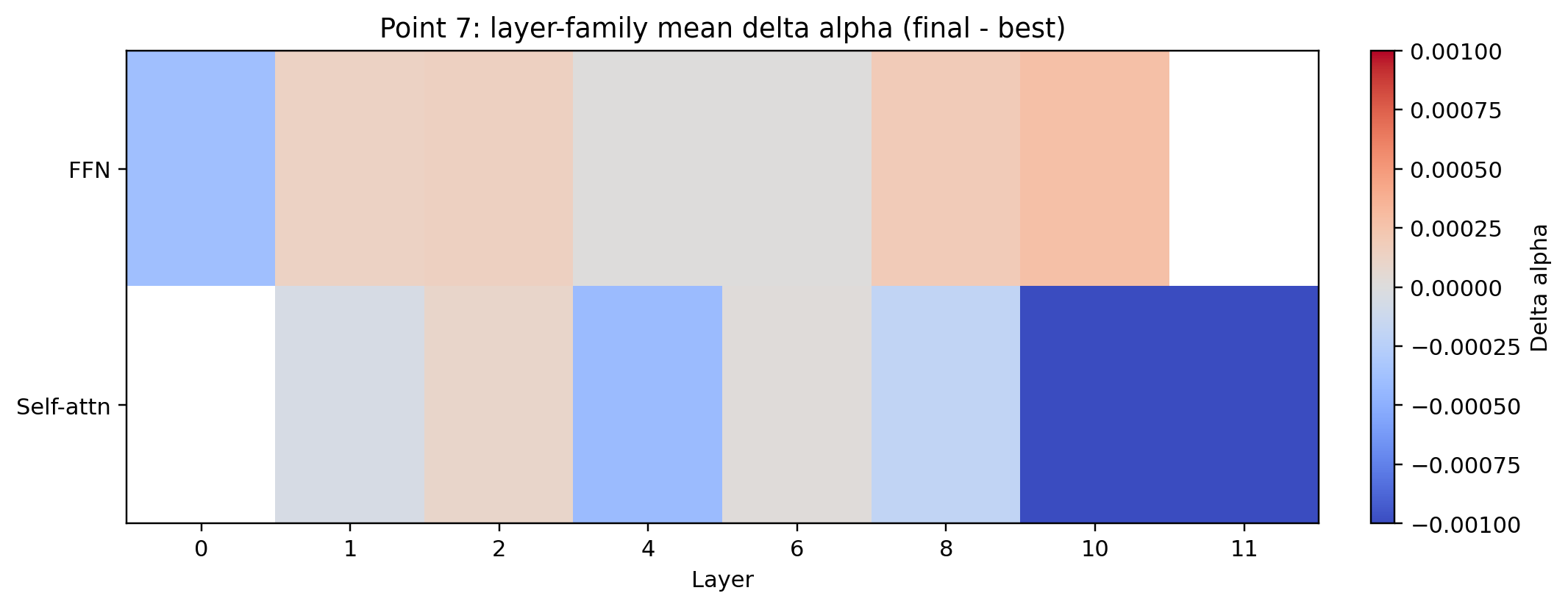

Supplement: Supplementary file 2 — Supporting File 2: advs76827‐sup‐0002‐DataSet.zip. [file ADVS-9999-e76827-s002.zip › outputs/reviewer_point7_grokking_esd/supplementary_point7/point7_supp_esd_delta_heatmap.png]

Point 7: Family-level ESD alpha stability

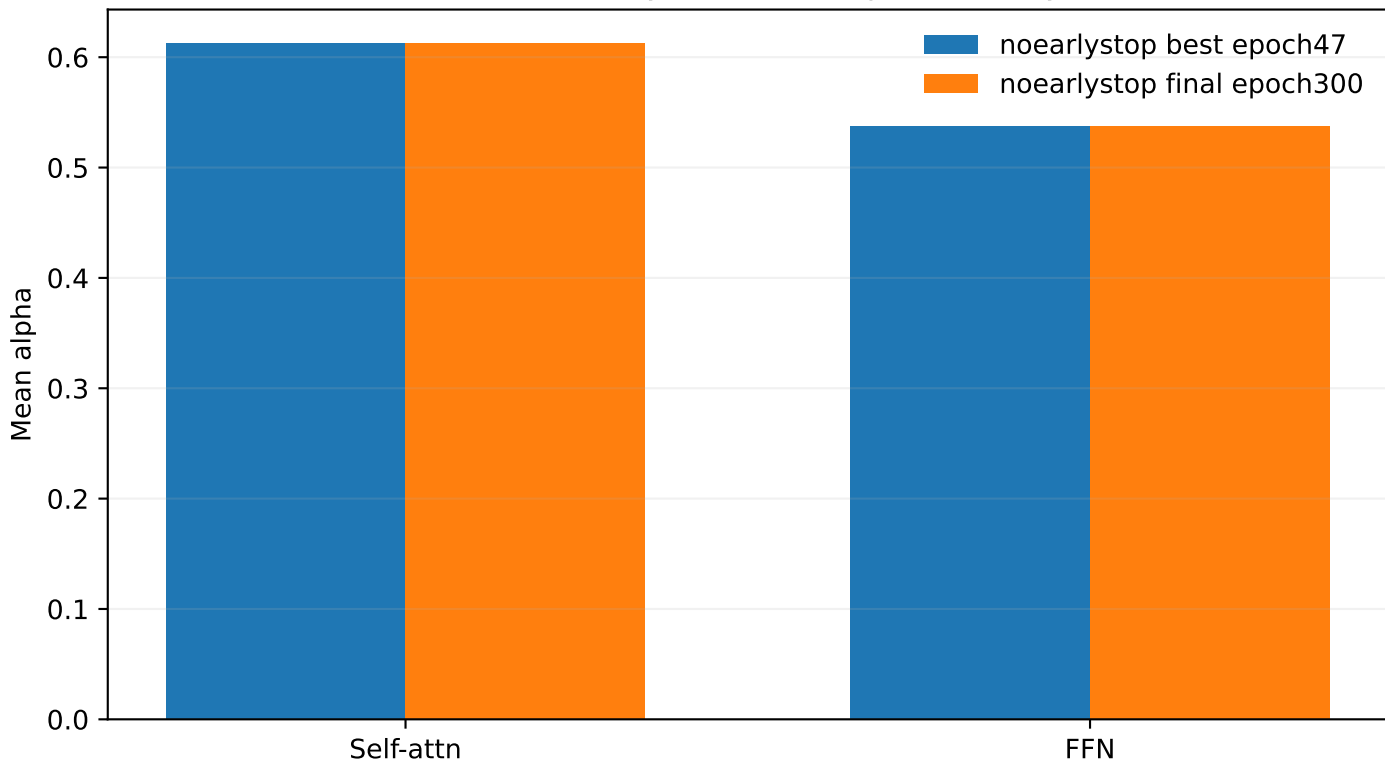

Supplement: Supplementary file 2 — Supporting File 2: advs76827‐sup‐0002‐DataSet.zip. [file ADVS-9999-e76827-s002.zip › outputs/reviewer_point7_grokking_esd/supplementary_point7/point7_supp_esd_family_alpha.pdf]

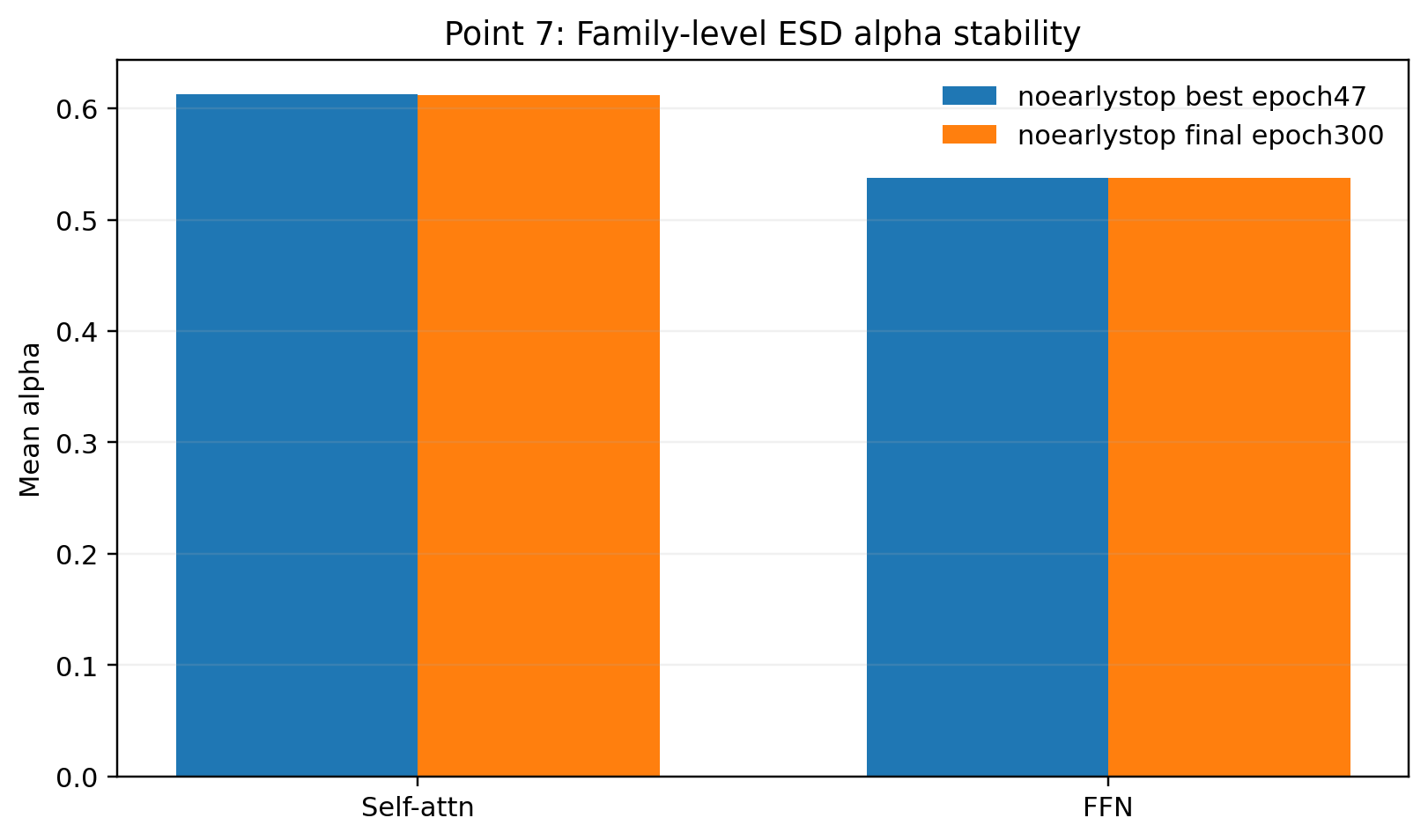

Supplement: Supplementary file 2 — Supporting File 2: advs76827‐sup‐0002‐DataSet.zip. [file ADVS-9999-e76827-s002.zip › outputs/reviewer_point7_grokking_esd/supplementary_point7/point7_supp_esd_family_alpha.png]

## Point 7: Random baseline post-69 dynamics

Full 300-epoch run

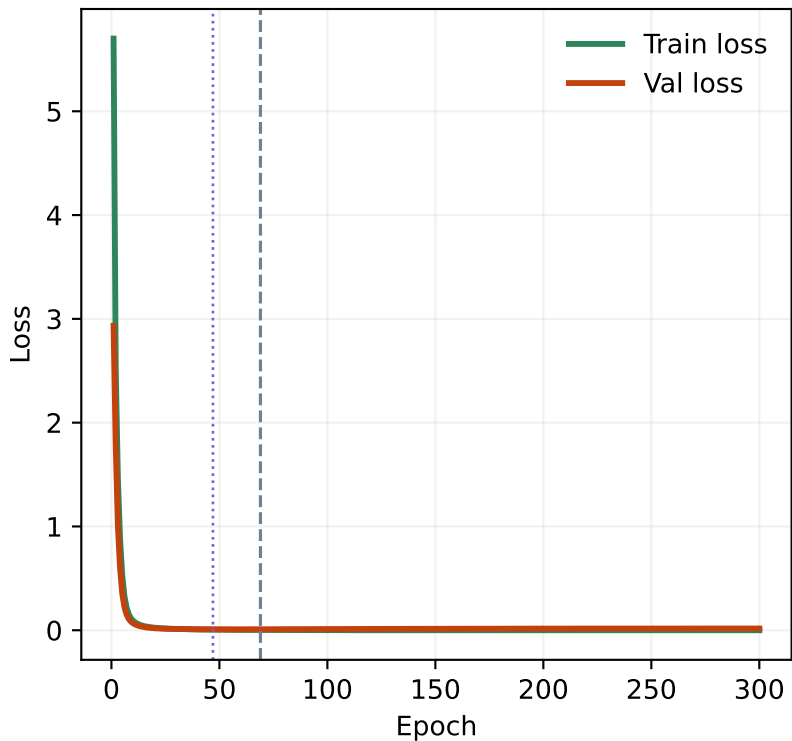

Post-69 zoom: train down, validation up

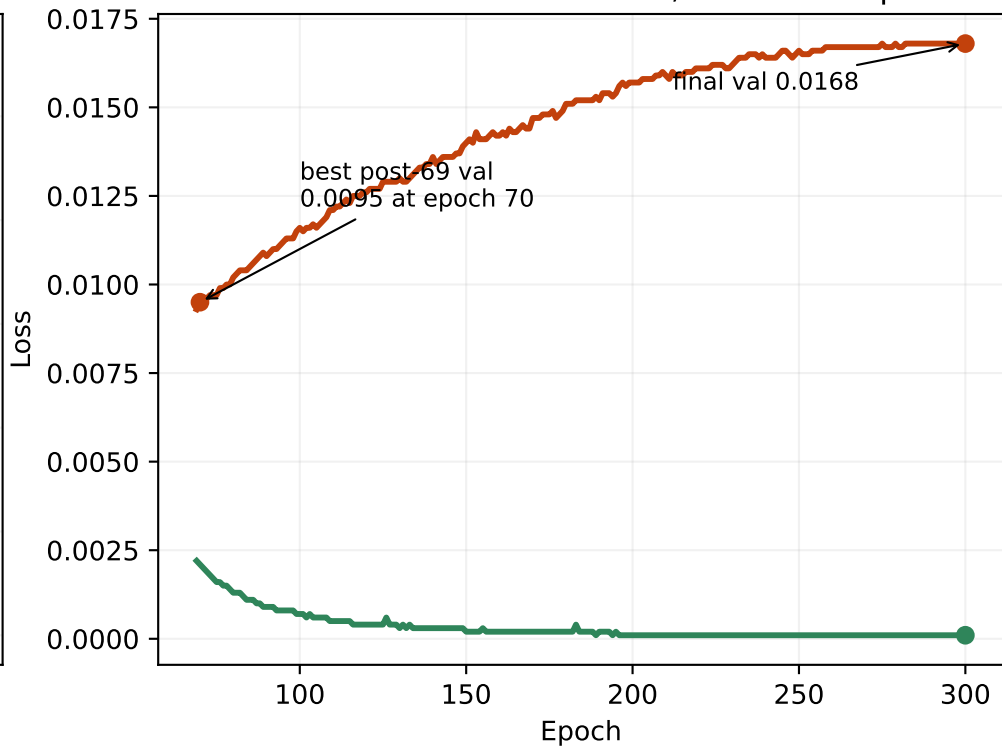

Supplement: Supplementary file 2 — Supporting File 2: advs76827‐sup‐0002‐DataSet.zip. [file ADVS-9999-e76827-s002.zip › outputs/reviewer_point7_grokking_esd/supplementary_point7/point7_supp_post69_dynamics.pdf]

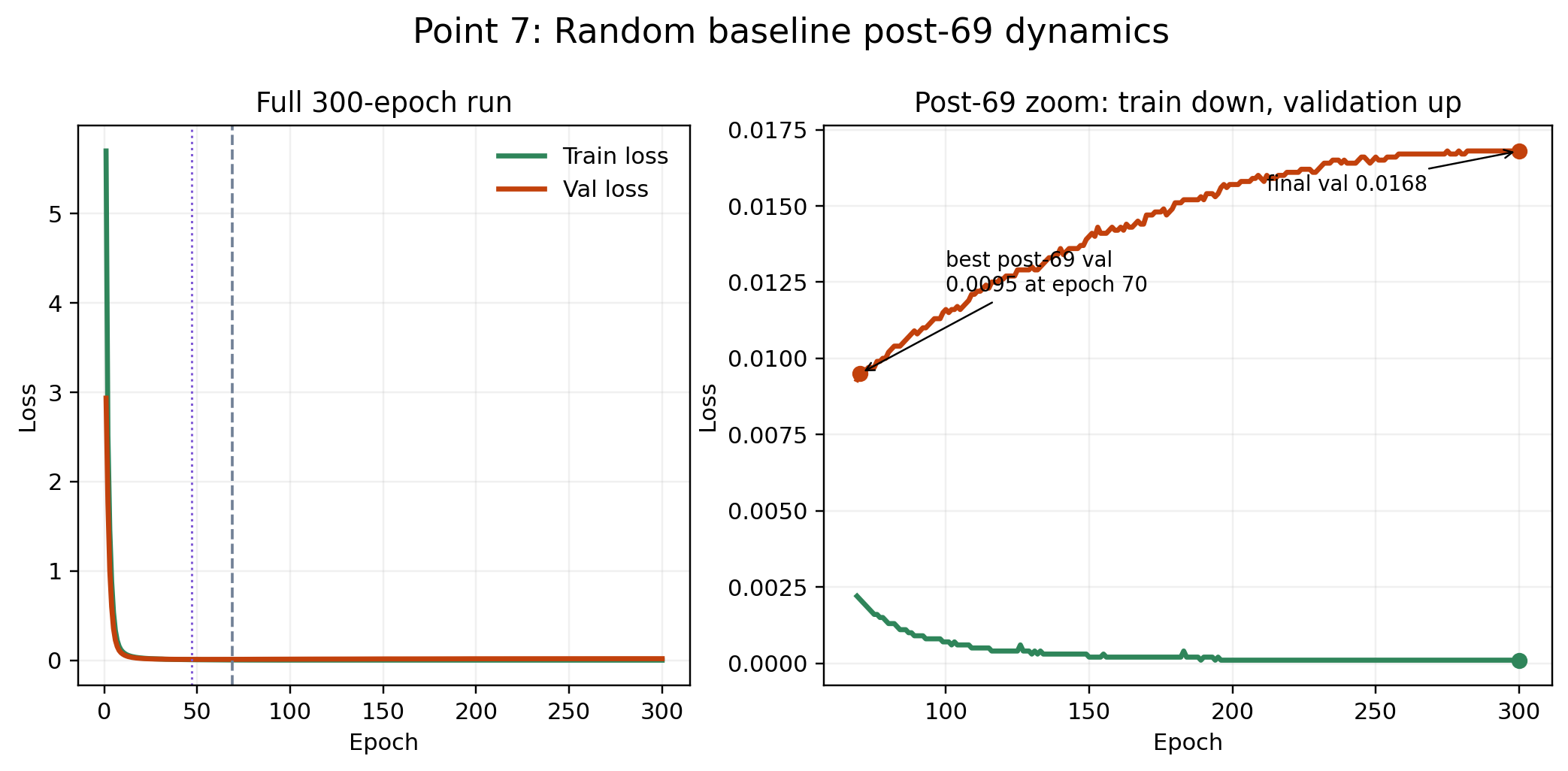

Supplement: Supplementary file 2 — Supporting File 2: advs76827‐sup‐0002‐DataSet.zip. [file ADVS-9999-e76827-s002.zip › outputs/reviewer_point7_grokking_esd/supplementary_point7/point7_supp_post69_dynamics.png]

Point 7: Random final drop is larger than the binomial CI

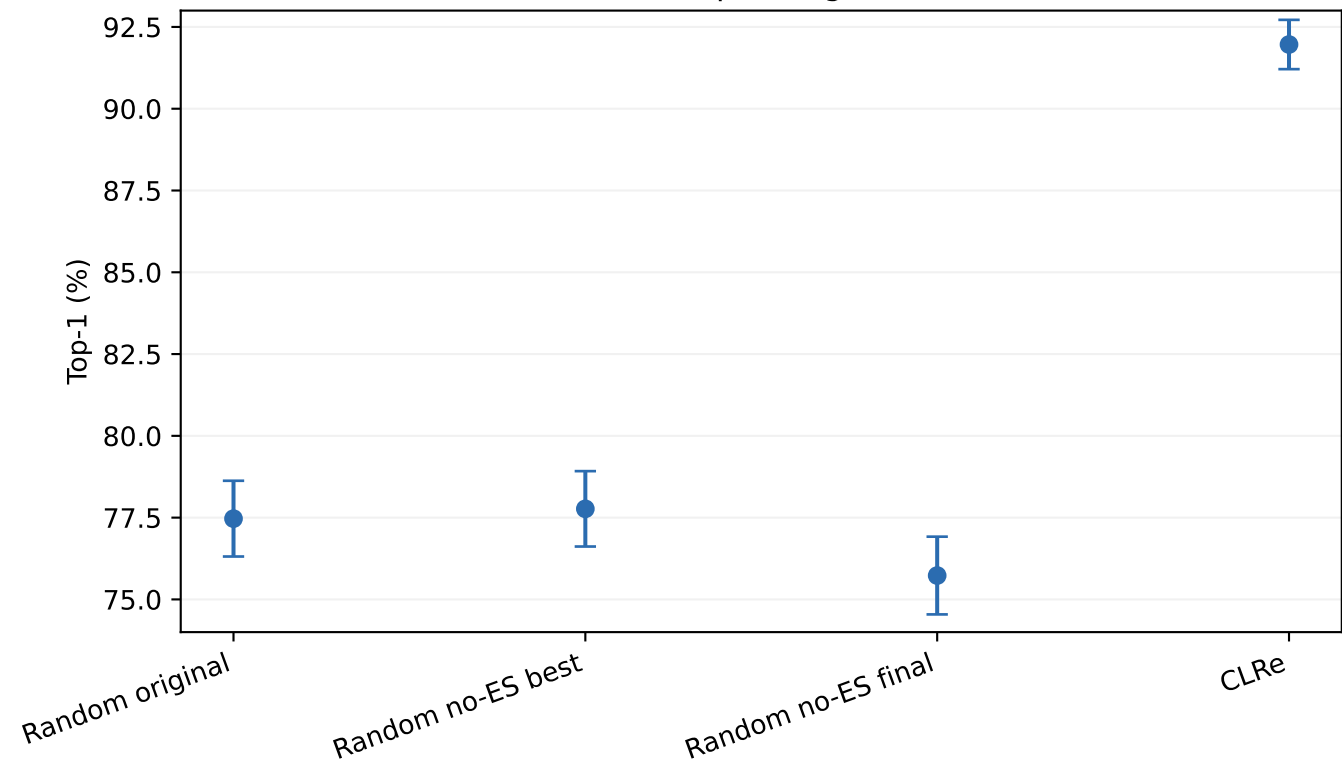

Supplement: Supplementary file 2 — Supporting File 2: advs76827‐sup‐0002‐DataSet.zip. [file ADVS-9999-e76827-s002.zip › outputs/reviewer_point7_grokking_esd/supplementary_point7/point7_supp_top1_ci.pdf]

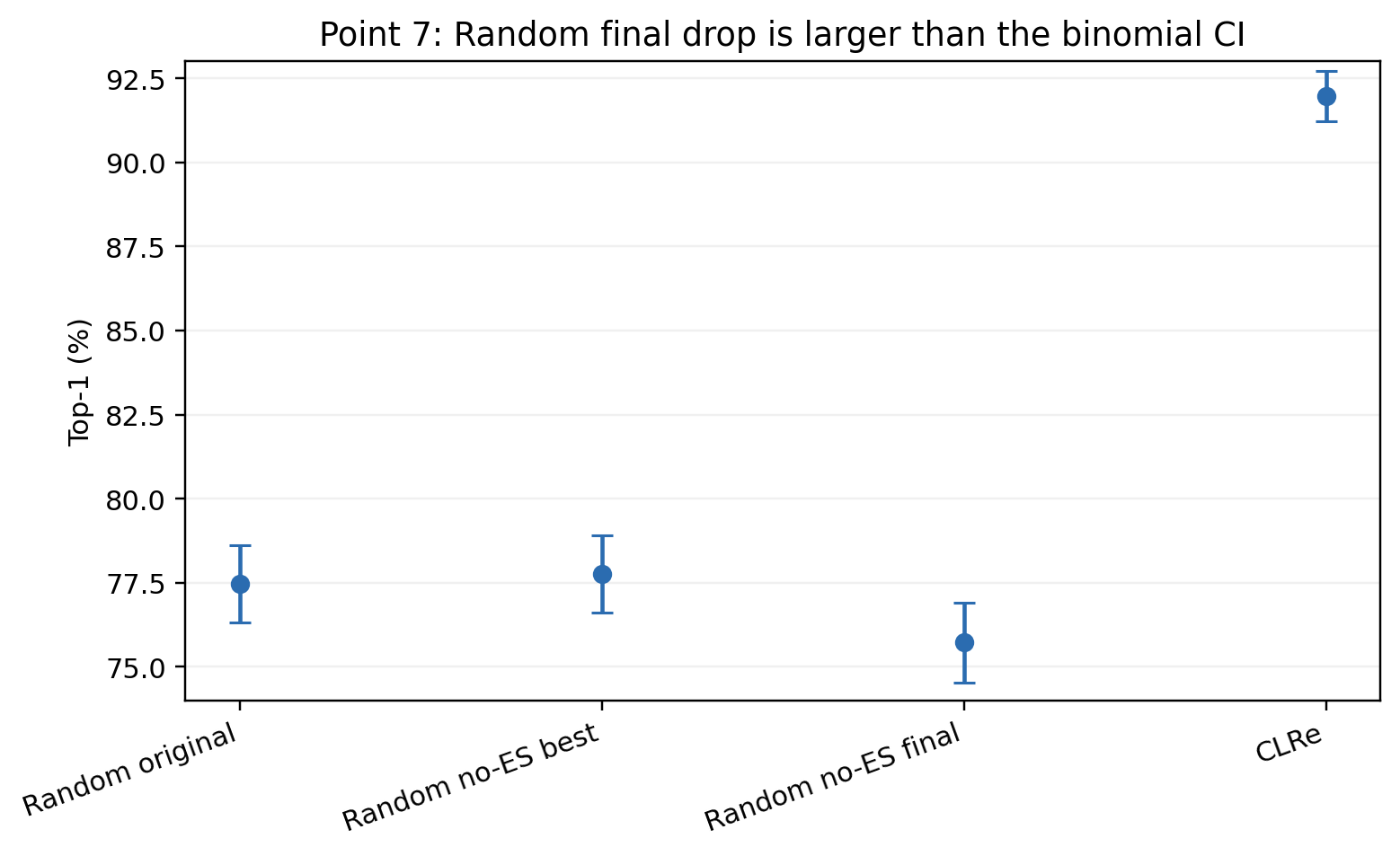

Supplement: Supplementary file 2 — Supporting File 2: advs76827‐sup‐0002‐DataSet.zip. [file ADVS-9999-e76827-s002.zip › outputs/reviewer_point7_grokking_esd/supplementary_point7/point7_supp_top1_ci.png]

## Point 7: top-k confidence intervals across all reported cutoffs

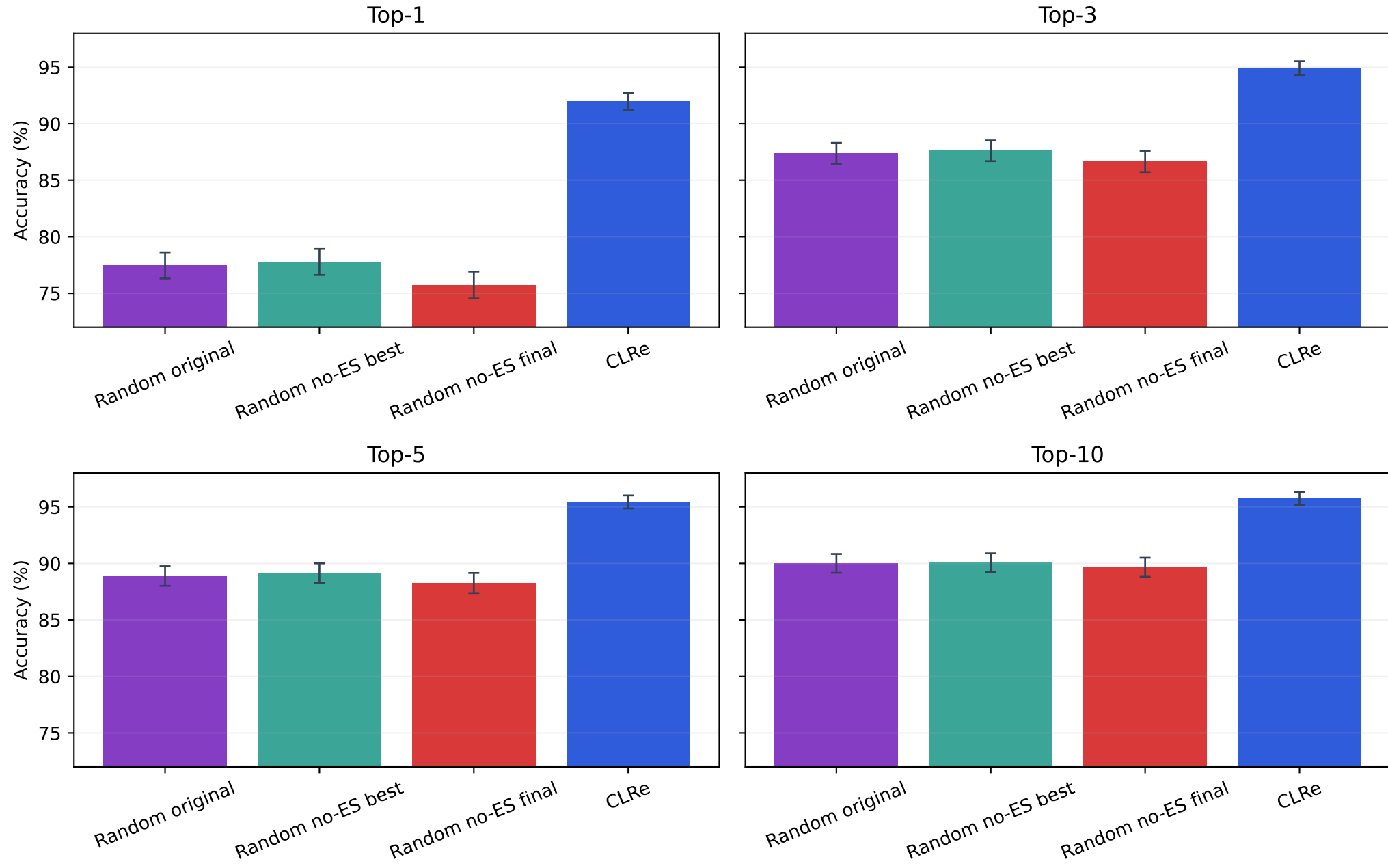

Supplement: Supplementary file 2 — Supporting File 2: advs76827‐sup‐0002‐DataSet.zip. [file ADVS-9999-e76827-s002.zip › outputs/reviewer_point7_grokking_esd/supplementary_point7/point7_supp_topk_panel.pdf]

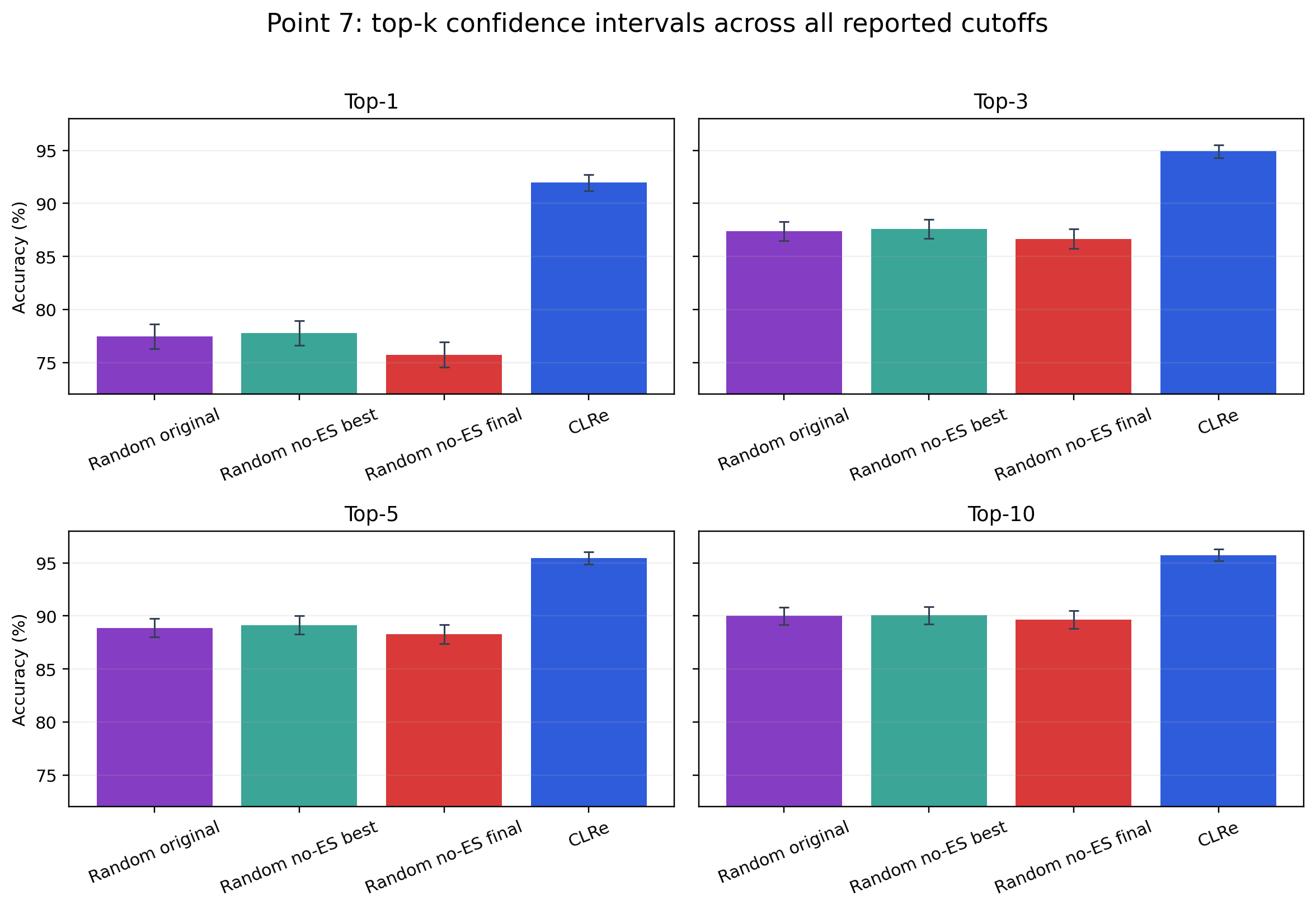

Supplement: Supplementary file 2 — Supporting File 2: advs76827‐sup‐0002‐DataSet.zip. [file ADVS-9999-e76827-s002.zip › outputs/reviewer_point7_grokking_esd/supplementary_point7/point7_supp_topk_panel.png]

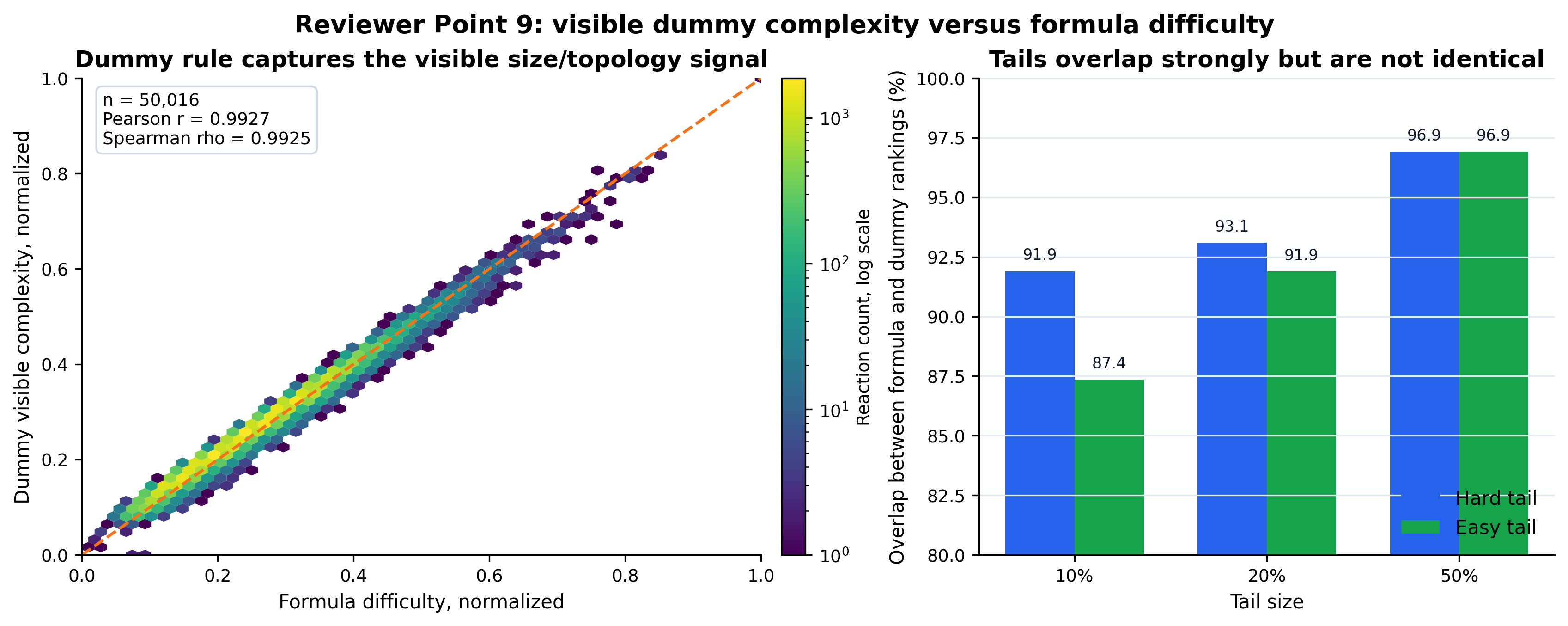

Supplement: Supplementary file 2 — Supporting File 2: advs76827‐sup‐0002‐DataSet.zip. [file ADVS-9999-e76827-s002.zip › outputs/reviewer_point9_dummy_complexity/figures/figure_point9_dummy_formula_relationship.png]
